# Hydropersulfides inhibit lipid peroxidation and ferroptosis by scavenging radicals

**DOI:** 10.1038/s41589-022-01145-w

**Published:** 2022-09-15

**Authors:** Uladzimir Barayeu, Danny Schilling, Mohammad Eid, Thamara Nishida Xavier da Silva, Lisa Schlicker, Nikolina Mitreska, Christopher Zapp, Frauke Gräter, Aubry K. Miller, Reinhard Kappl, Almut Schulze, José Pedro Friedmann Angeli, Tobias P. Dick

**Affiliations:** 1 Division of Redox Regulation, German Cancer Research Center (DKFZ), Heidelberg, Germany; 2 Faculty of Biosciences, Heidelberg University, Heidelberg, Germany; 3 Rudolf-Virchow-Zentrum - Center for Integrative and Translational Bioimaging, University of Würzburg, Würzburg, Germany; 4 Division of Tumor Metabolism and Microenvironment, German Cancer Research Center (DKFZ), Heidelberg, Germany; 5 Proteomics Core Facility, German Cancer Research Center (DKFZ), Heidelberg, Germany; 6 Department of Biophysics, Faculty of Medicine, Center for Integrative Physiology and Molecular Medicine (CIPMM), Saarland University, Homburg, Germany; 7 Molecular Biomechanics, Heidelberg Institute for Theoretical Studies (HITS), Heidelberg, Germany; 8 Institute for Theoretical Physics, Heidelberg University, Heidelberg, Germany; 9 Research Group Cancer Drug Development, German Cancer Research Center (DKFZ), Heidelberg, Germany

**Keywords:** Cell death, Metabolic pathways, Biophysical chemistry, Lipids

## Abstract

Ferroptosis is a type of cell death caused by radical-driven lipid peroxidation, leading to membrane damage and rupture. Here we show that enzymatically produced sulfane sulfur (S^0^) species, specifically hydropersulfides, scavenge endogenously generated free radicals and, thereby, suppress lipid peroxidation and ferroptosis. By providing sulfur for S^0^ biosynthesis, cysteine can support ferroptosis resistance independently of the canonical GPX4 pathway. Our results further suggest that hydropersulfides terminate radical chain reactions through the formation and self-recombination of perthiyl radicals. The autocatalytic regeneration of hydropersulfides may explain why low micromolar concentrations of persulfides suffice to produce potent cytoprotective effects on a background of millimolar concentrations of glutathione. We propose that increased S^0^ biosynthesis is an adaptive cellular response to radical-driven lipid peroxidation, potentially representing a primordial radical protection system.

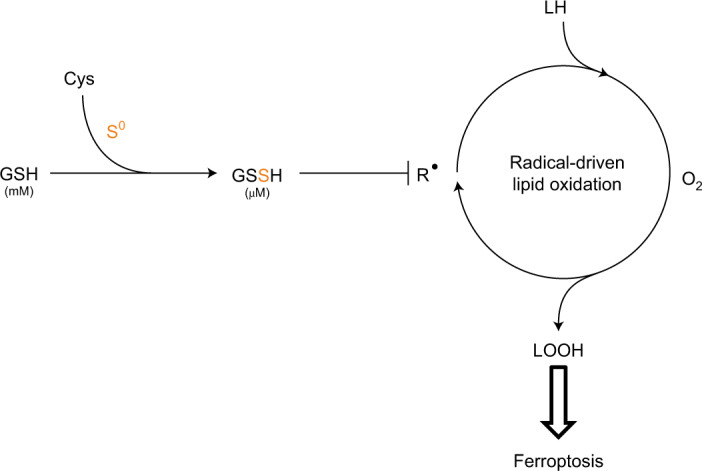

## Abstract (web summary)

Enzymatically generated sulfane sulfur species called hydropersulfides terminate free radical chain reactions to prevent oxidative membrane damage and ferroptosis induction.

## Main

Ferroptosis is caused by membrane lipid peroxidation (LPO)^1^. Low rates of LPO are under cellular control and part of normal physiology. However, when some threshold of LPO is reached, control is lost, and ferroptosis takes place. LPO can get out of control, because it can be propagated by a radical chain reaction^2^. In essence, radical reactions circumvent the spin barrier limiting triplet oxygen reactivity, thus driving thermodynamically favorable lipid oxidation, leading to the disruption of membrane integrity and function.

Although ferroptosis does not seem to be ‘programmed’, it is tightly controlled in the sense of being enabled or limited, depending on the presence or absence of various promoting or inhibiting factors. These factors include (1) the amount of polyunsaturated fatty acids integrated into phospholipids^3,4^; (2) iron metabolism and compartmentalization^5^; (3) enzymatic LPO—that is, lipoxygenase activity^6^; (4) the presence of membrane-associated radical scavengers, such as ɑ-tocopherol, CoQ_10_ (refs. ^7,8^) or tetrahydrobiopterin^9^; and (5) the cysteine (Cys)→glutathione (GSH)→glutathione peroxidase 4 (GPX4) axis, facilitating reduction of lipid hydroperoxides to lipid alcohols^3^. Numerous auxiliary and supply pathways converge on this axis—for example, provisioning of Cys for GSH synthesis^10^, NADPH-dependent glutathione reduction and selenium incorporation into GPX4 (ref. ^11^). More recently, it has become clear that most cells are highly flexible in employing different mechanisms by which they avoid excess LPO and ferroptosis. Tumor cells are often more susceptible to ferroptosis but, in many cases, can compensate for the inhibition of individual ferroptosis-preventing pathways^12,13^. Furthermore, results from genetic screening suggest that anti-ferroptotic strategies are highly cell type specific and often context dependent^9^. Thus, there are strong indications that additional anti-ferroptotic strategies remain to be discovered.

In this study, we explored the possible connection among S^0^ biology, LPO and ferroptosis. The biology of S^0^ species (that is, hydropersulfides, hydropolysulfides and polysulfides) is a relatively new field of study. Nevertheless, it is now well-established that all cells can produce persulfides and polysulfides endogenously, through several enzymatic pathways and mechanisms, Cys being the ultimate source of sulfur^14^. Previous studies have indicated that S^0^ species can have potent anti-oxidative and cytoprotective properties^15,16^. These studies have mostly focused on the reactions between S^0^ species and two-electron oxidants. However, a puzzling aspect of S^0^ biology is the low intracellular concentration of persulfides and polysulfides (low micromolar range)^15^ relative to glutathione (millimolar range), which makes it difficult to rationalize an efficient scavenging role for S^0^ species. Furthermore, the influence of biological S^0^ species on one-electron oxidants (radicals) has so far received little attention. Although it has been shown in earlier studies that polysulfides can prevent lipid oxidation in vitro^17^, the prevailing view has been that persulfides and polysulfides react with product peroxides, thus preventing the initiation of new chain reactions. However, work in the 1990s and during the last few years has shown that hydropersulfides are excellent hydrogen atom transfer agents, directly engaging in one-electron reactions^18–20^, although the exact mechanisms and their relevance to biological systems remain to be clarified.

Here we show that biologically relevant S^0^ species are potent radical scavengers and chain terminators, in vitro and inside living cells, and are important for the protection of membranes against LPO and, thus, are also relevant to ferroptosis sensitivity. Specifically, we report the following. (1) Pro-ferroptotic conditions trigger an increase in intracellular S^0^ levels, likely constituting an adaptive cellular response. (2) Increased cellular uptake of Cys can protect against ferroptosis independently of GPX4 by supplying sulfur to S^0^ biosynthetic pathways. (3) The elevation of endogenous S^0^ levels makes cells more resistant to LPO and ferroptosis, whereas the lowering of S^0^ levels sensitizes cells to LPO and ferroptosis. (4) An increase of endogenous S^0^ levels lowers endogenous radical load, and a decrease of endogenous S^0^ levels increases endogenous radical load. (5) Exogenously supplied S^0^ donors suppress ferroptosis in various cellular models. (6) Persulfides catalyze GSH-dependent radical scavenging in vitro, as they are regenerated via an autocatalytic cycle that couples radical reduction to glutathione oxidation through the formation and recombination of perthiyl radicals. Together, these results establish hydropersulfides as important radical scavengers and modulators of ferroptotic cell death.

## Results

### Cystine uptake can inhibit ferroptosis independently of GPX4

**Fig. 1:**
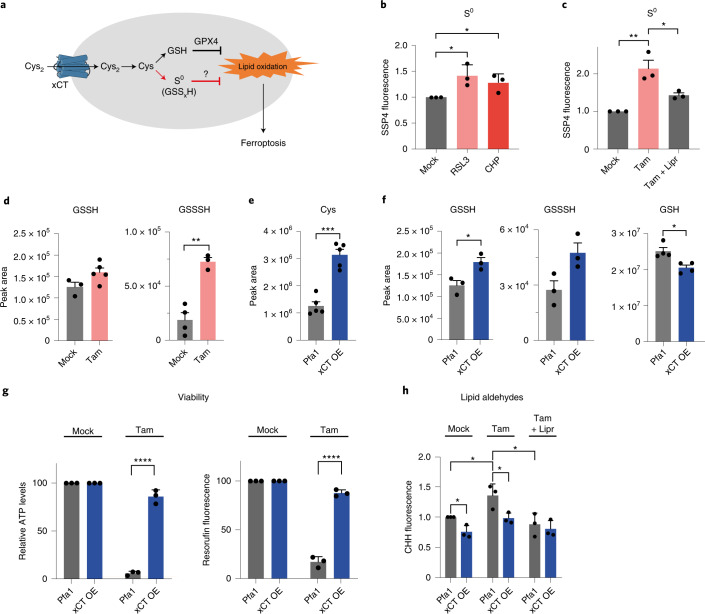
Cys uptake can inhibit ferroptosis independently of GPX4. **a**, Pathways by which cellular Cys_2_ uptake may contribute to ferroptosis resistance. On the one hand, Cys serves the biosynthesis of GSH, which is required by GPX4 to reduce oxidized lipids (upper branch). On the other hand, Cys is a source of S^0^ species, which may counteract lipid oxidation in a GPX4-independent manner (lower branch). **b**, S^0^ levels measured by SSP4 staining 4 hours after treatment of HeLa cells with either RSL3 (5 µM) or CHP (40 µM). *n* = 3. *P* = 0.0282 and 0.0500. **c**, S^0^ levels measured by SSP4 staining 48 hours after the addition of 4-hydroxytamoxifen (Tam, 0.75 µM) and liproxstatin-1 (Lipr, 1 µM) to Pfa1 cells. *n* = 3. *P* = 0.0070 and 0.0377. **d**, GSSH/GSSSH levels measured by LC–MS 24 hours after the addition of 4-hydroxytamoxifen (Tam, 0.75 µM) to Pfa1 cells. Left panel: *n* = 3 (mock), *n* = 5 (Tam), *P* = 0,0754; right panel: *n* = 4 (mock), *n* = 3 (Tam), *P* = 0.0015. **e**, Cys levels as measured by LC–MS in Pfa1 and xCT OE cells. *n* = 5. *P* = 0.00007. **f**, GSSH, GSSSH and GSH levels in Pfa1 and xCT OE cells as measured by LC–MS. Left panel: *n* = 3, *P* = 0.0251; middle panel: *n* = 3, *P* = 0.0504; right panel: *n* = 4, *P* = 0.0103. **g**, Cell viability after 72 hours of incubation with 4-hydroxytamoxifen (Tam, 0.5 µM), measured with an ATP assay (left panel) and a PrestoBlue (reduction capacity) assay (right panel) in Pfa1 and xCT OE cells. Left panel: *n* = 3, *P* = 0.00005; right panel: *n* = 3, *P* = 0.00004. **h**, Lipid aldehyde levels in Pfa1 and xCT OE cells, 48 hours after the addition of tamoxifen (Tam, 0.75 µM), as measured by CHH staining. *n* = 3. *P* = 0.0157, 0.0370, 0.0404 and 0.0397. Data are presented as mean values. For LC–MS data, error bars represent s.e.m. For the rest, error bars represent s.d. * *P* ≤ 0.05; ** *P* ≤ 0.01; *** *P* ≤ 0.001; and **** *P* ≤ 0.0001 based on a two-tailed unpaired *t*-test. OE, overexpressing. Source data

**Extended Data Fig. 1:**
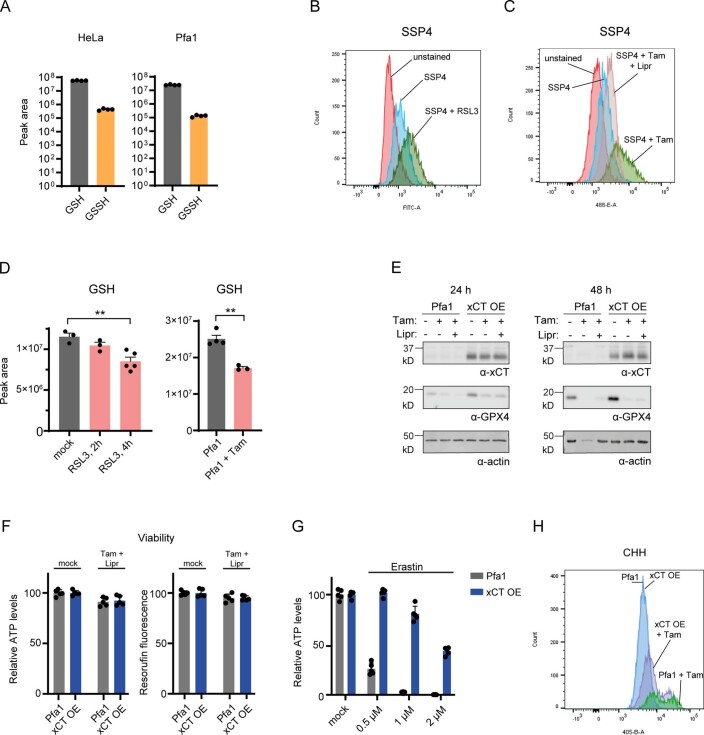
Cysteine rescues cells from ferroptosis independently of GPX4. (**A**) Relative GSH and GSSH levels in HeLa and Pfa1 cells as measured by LC-MS. n = 4. (**B**) Flow cytometry of SSP4 stained HeLa cells treated with RSL3 (5 µM) for 4 hours. (**C**) Flow cytometry of SSP4 stained Pfa1 cells treated with tamoxifen (Tam; 0.75 µM), in the presence or absence of liproxstatin-1 (Lipr; 1 µM), for 48 hours. (**D**) GSH levels in HeLa cells treated with RSL3 (5 µM) (left panel) and in Pfa1 cells treated with Tam (0.75 µM) for 24 h (right panel), as measured by LC-MS. Left panel: n = 3, p = 0.1379 (mock, RSL3 2 h). n = 5, p = 0.0067 (RSL3 4 h). Right panel: n = 4 (Pfa1), n = 3, p = 0.0014 (Tam). (**E**) Immunoblotting of xCT, GPX4 and α-actin in Pfa1 and xCT OE cells, 24 h (left panel) or 48 h (right panel) after treatment with Tam (0.75 µM) and/or Lipr (1 µM). (**F**) Viability of Pfa1 and xCT OE cells after 72 h of incubation with Tam (0.5 µM) and Lipr (1 µM), as measured with an ATP assay (left panel) and a PrestoBlue cell viability assay (right panel). n = 5. (**G**) Viability of Pfa1 and xCT OE cells after 72 h of incubation with erastin, as measured with an ATP assay. n = 5. (**H**) Flow cytometry of coumarin hydrazide (CHH) stained Pfa1 and xCT OE cells treated with Tam (0.75 µM) for 48 h. Data are presented as mean values. For LC/MS data error bars represent SEM. For the rest, error bars represent SD, ** *P* ≤ 0.01 based on a two-tailed unpaired *t*-test. Source data

It is well-understood that cellular cystine (Cys_2_) uptake contributes to ferroptosis resistance. Inside the cell, Cys_2_ is quickly reduced to Cys. Cys is needed for the synthesis of GSH, which, in turn, allows GPX4 to reduce lipid peroxides. However, it has been shown more recently that Cys is also a source of sulfur for S^0^ species, including glutathione hydropersulfide and hydropolysulfides (GSS_x_H)^14,21^. Analyzing the cells used in this study, we found that about 1% of the total GSH pool is persulfidated (Extended Data Fig. 1a). We, therefore, asked if S^0^ could contribute to ferroptosis resistance by suppressing LPO in a GPX4-independent manner (Fig. 1a). To address this question, we first assessed how endogenous S^0^ levels change when cells are primed to undergo ferroptosis. Treatment of cells with the GPX4 inhibitor RSL3 or the lipophilic oxidant cumene hydroperoxide (CHP) increased endogenous S^0^ levels (Fig. 1b and Extended Data Fig. 1b). Similarly, genetic deletion of floxed *Gpx4* in Pfa1 cells^22^ increased endogenous S^0^ levels, including glutathione hydropersulfide and hydrotrisulfide (GSSH and GSSSH, respectively) (Fig. 1c,d and Extended Data Fig. 1c). At the same time, inhibition or deletion of GPX4 led to a decrease in GSH levels (Extended Data Fig. 1d), indicating that cells consume GSH upon ferroptosis induction. We then asked if increased intracellular Cys availability could inhibit ferroptosis even in the absence of GPX4. To this end, we overexpressed the murine cystine/glutamate antiporter xCT (Slc7a11) in Pfa1 cells (Extended Data Fig. 1e). As expected, xCT overexpression increased intracellular Cys levels (Fig. 1e). Interestingly, xCT overexpression also led to an increase in GSSH and GSSSH and to a decrease in GSH (Fig. 1f). When we combined xCT overexpression with GPX4 deletion, we found that elevated Cys availability rescues GPX4-deficient cells from ferroptotic cell death (Fig. 1g). As expected, the death of non-xCT-overexpressing cells was almost completely rescued by the lipophilic radical scavenger liproxstatin-1 (Extended Data Fig. 1f), and rescue by xCT overexpression was countermanded by the xCT inhibitor erastin (Extended Data Fig. 1g). Moreover, xCT overexpression lowered endogenous lipid aldehyde levels both before and after GPX4 deletion (Fig. 1h and Extended Data Fig. 1h), indicative of a decreased LPO rate. Together, these findings support the conclusion that Cys has anti-ferroptotic effects beyond its use as a building block for the GPX4 cofactor GSH. Furthermore, they suggest that Cys-dependent biosynthesis of S^0^ could be an important adaptive cellular response to pro-ferroptotic conditions.

### S^0^-generating/degrading enzymes modulate LPO and ferroptosis

**Extended Data Fig. 2:**
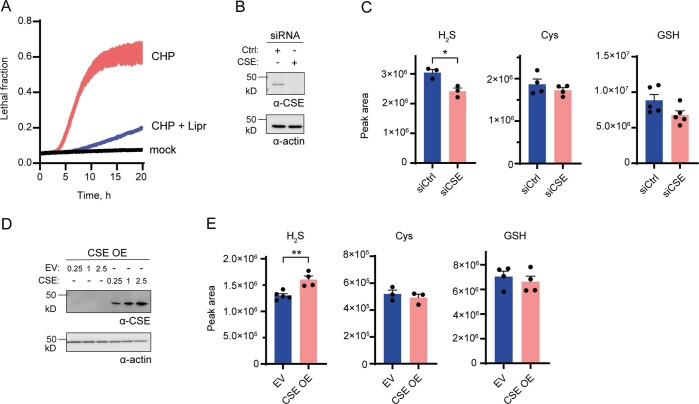
CSE knockdown and overexpression. (**A**) Loss of viability in HeLa cells treated with CHP (40 µM), in the presence or absence of liproxstatin-1 (Lipr, 20 µM), measured with the CellTox Green cytotoxicity assay. n = 3. (**B**) Depletion of CSE in HeLa cells, demonstrated by immunoblotting. (**C**) Influence of depleting CSE (siCSE) on H_2_S, Cys and GSH levels in HeLa cells, as measured by LC-MS. Left panel: n = 3, p = 0.0143; Middle panel: n = 4, p = 0.3399; Right panel: n = 5, p = 0.0753. (**D**) Overexpression of CSE in HeLa cells, demonstrated by immunoblotting. **(E)** Influence of overexpressing CSE (CSE OE) on H_2_S, Cys and GSH levels in HeLa cells, as measured by LC-MS. EV: empty vector. Left panel: n = 5 (EV), n = 4 (CSE OE), p = 0.0038; Middle panel: n = 3, p = 0.8181; Right panel: n = 5, p = 0.5282. Data are presented as mean values. Error bars represent SEM, * *P* ≤ 0.05; ** *P* ≤ 0.01 based on a two-tailed unpaired *t*-test. Source data

Next, we asked if ferroptosis sensitivity could be modulated by enzymes involved in the generation and degradation of S^0^ species. To this end, we genetically manipulated the expression levels of key enzymes in HeLa cells. To assess the effect of these manipulations, we monitored the loss of membrane integrity under conditions of ferroptosis induction. As expected, the loss of membrane integrity in wild-type HeLa cells upon treatment with RSL3 or CHP was mostly prevented by liproxstatin-1 (Extended Data Fig. 2a). We first modulated levels of the H_2_S-generating enzyme cystathionine γ-lyase (CSE, also known as CTH). As expected, the depletion of CSE (Extended Data Fig. 2b) lowered endogenous H_2_S levels (Extended Data Fig. 2c), and the overexpression of CSE (Extended Data Fig. 2d) increased endogenous H_2_S levels (Extended Data Fig. 2e).

**Fig. 2:**
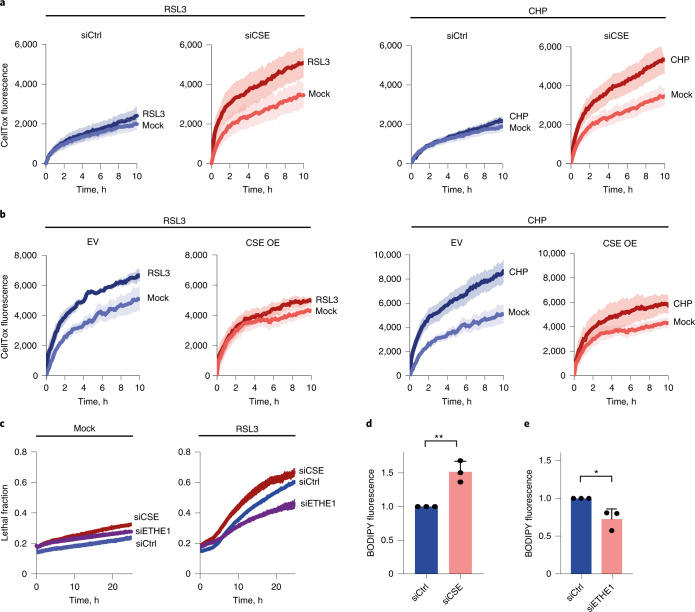
S^0^-generating/degrading enzymes modulate LPO and ferroptosis. **a**, Influence of depleting CSE (siCSE) on the loss membrane integrity triggered by treatment of HeLa cells with either RSL3 (5 µM) (left two panels) or CHP (40 µM) (right two panels), as measured by the CellTox Green cytotoxicity assay. *n* = 3. **b**, Influence of overexpressing CSE (CSE OE) on the loss of membrane integrity triggered by treatment of HeLa cells with either RSL3 (5 µM) (left two panels) or CHP (40 µM) (right two panels), as measured by the CellTox Green cytotoxicity assay. *n* = 3. **c**, Influence of depleting CSE (siCSE) or ETHE1 (siETHE1) on the loss membrane integrity triggered by treatment of HeLa cells with RSL3 (5 µM), as measured by the CellTox Green cytotoxicity assay. Fluorescence is normalized to the lysis control (lethal fraction). *n* = 3. **d**, Influence of depleting CSE (siCSE) on the oxidation of BODIPY-C11, as induced by incubation with RSL3 (5 µM) for 4 hours. *n* = 3. *P* = 0.0049. **e**, Influence of depleting ETHE1 (siETHE1) on the oxidation of BODIPY-C11, as induced by incubation with RSL3 (2 µM) for 3 hours. *n* = 3. *P* = 0.0236. Data are presented as mean values. For CellTox assays, error bars represent s.e.m. For the rest, error bars represent s.d. * *P* ≤ 0.05 and ** *P* ≤ 0.01 based on a two-tailed unpaired *t*-test. Source data

**Extended Data Fig. 3:**
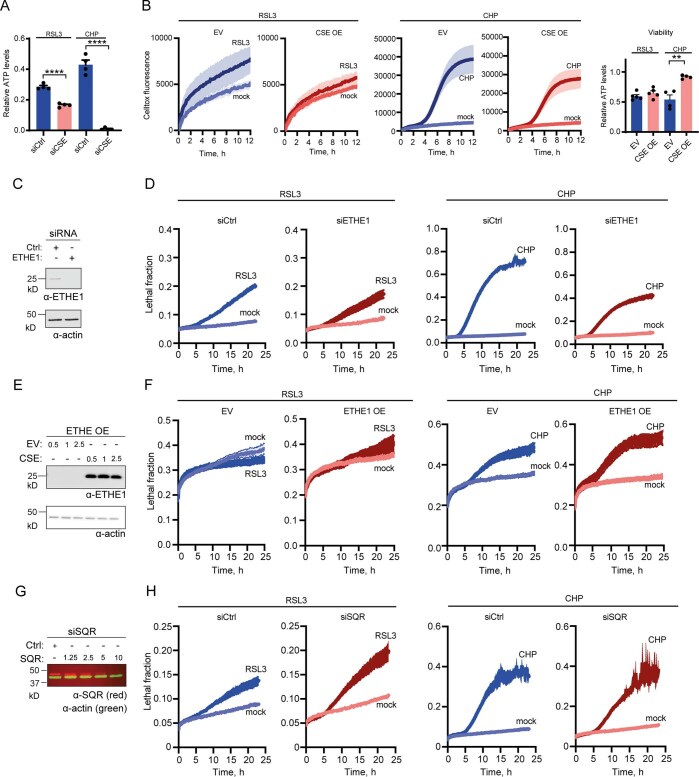
Influence of S^0^ metabolizing enzymes on membrane integrity. (**A**) Influence of CSE depletion on the viability of HeLa cells treated for 24 h with either RSL3 (5 µM) or CHP (40 µM), as measured with an ATP assay. n = 4. p = 0.00003 (RSL3), p = 0.00001 (CHP). (**B**) Influence of CSE overexpression (OE) on the loss of viability of U2OS cells treated with either RSL3 (5 µM) (left panels) or CHP (40 µM) (right panels), measured with the CellTox Green cytotoxicity assay. Rightmost panel: Influence of CSE overexpression (OE) on the viability of U2OS cells treated for 24 h with either RSL3 (5 µM) or CHP (40 µM), as measured with an ATP assay. EV: empty vector. n = 3. Right panel: n = 4, p = 0.2763 (RSL3); n = 5, p = 0.0029 (CHP). (**C**) Depletion of ETHE1 in HeLa cells, demonstrated by immunoblotting. (**D**) Influence of ETHE1 depletion on the loss of viability of HeLa cells treated with either RSL3 (5 µM) (left panels) or CHP (40 µM) (right panels), measured with the CellTox Green cytotoxicity assay. n = 3. (**E**) Overexpression of ETHE1 in HeLa cells, demonstrated by immunoblotting. (**F**) Influence of ETHE1 overexpression on the loss of viability of HeLa cells treated with either RSL3 (5 µM) (left panels) or CHP (40 µM) (right panels), measured with the CellTox Green cytotoxicity assay. n = 3. (**G**) Depletion of SQR in HeLa cells, as demonstrated by fluorescent antibody labeling. (**H**) Influence of SQR depletion on the loss of viability of HeLa cells treated with either RSL3 (5 µM) (left panels) or CHP (40 µM) (right panels), measured with the CellTox Green cytotoxicity assay. n = 3. Data are presented as mean values. For CellTox assay error bars represent SEM. For the rest, ** *P* ≤ 0.01; **** *P* ≤ 0.0001 based on a two-tailed unpaired *t*-test. Source data

We then found that downregulation of CSE sensitized cells to ferroptosis (Fig. 2a and Extended Data Fig. 3a), whereas its upregulation made cells more resistant (Fig. 2b). Similar results were obtained with an independent cell line (U2OS) (Extended Data Fig. 3b). Moreover, neither depletion nor overexpression of CSE significantly affected overall Cys and GSH levels (Extended Data Fig. 2c,e). Next, we modulated levels of the persulfide-degrading enzyme persulfide dioxygenase (ETHE1). A direct side-by-side comparison of CSE and ETHE1 depletion revealed opposing effects on ferroptosis sensitivity (Fig. 2c). Depletion of ETHE1 (Extended Data Fig. 3c) lowered ferroptosis sensitivity (Extended Data Fig. 3d), whereas its overexpression (Extended Data Fig. 3e) increased ferroptosis sensitivity (Extended Data Fig. 3f). Depletion of the persulfide-generating enzyme sulfide:quinone reductase (SQR) (Extended Data Fig. 3g) increased ferroptosis sensitivity (Extended Data Fig. 3h).

**Extended Data Fig. 4:**
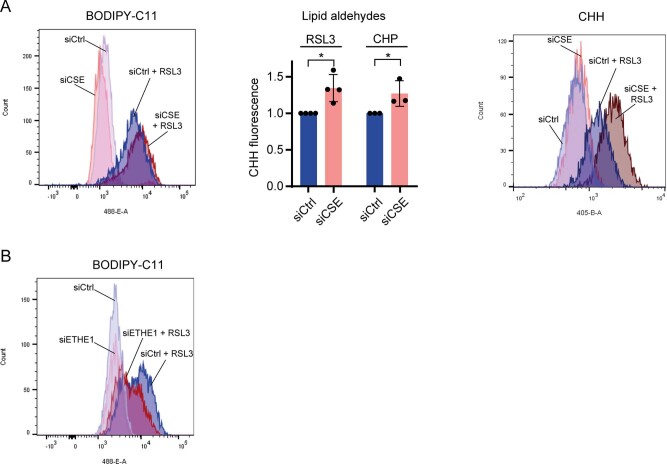
Influence of CSE and ETHE1 depletion on lipid oxidation. (**A**) Flow cytometry of BODIPY-C11 stained CSE-depleted HeLa cells following treatment with RSL3 (5 µM) for 4 h (left panel). Lipid aldehyde levels in CSE-depleted HeLa cells following treatment with either RSL3 (5 µM) or CHP (40 µM) for 4 h, as measured by CHH staining (middle panel). Flow cytometry of CHH stained CSE-depleted HeLa cells following treatment with RSL3 (5 µM) for 4 h (right panel). Middle panel: n = 4, p = 0.0101 (RSL3); n = 3, p = 0.05 (CHP). (**B**) Flow cytometry of BODIPY-C11 stained ETHE1-depleted HeLa cells following treatment with RSL3 (2 µM) for 3 h. Data are presented as mean values. Error bars represent SD, * *P* ≤ 0.05 based on a two-tailed unpaired *t*-test. Source data

Having observed the influence of S^0^-metabolizing enzymes on ferroptotic cell death, we next asked about their influence on LPO. To this end, we induced ferroptosis with RSL3 and measured LPO using BODIPY-C11 and coumarin hydrazide (CHH). Depletion of CSE elevated RSL3-induced LPO (Fig. 2d and Extended Data Fig. 4a), whereas depletion of ETHE1 lowered it (Fig. 2e and Extended Data Fig. 4b). Taken together, these results suggest that endogenously produced persulfides protect against LPO and consequently prevent ferroptosis.

### Exogenously supplied S^0^ eliminates intracellular radicals

**Fig. 3:**
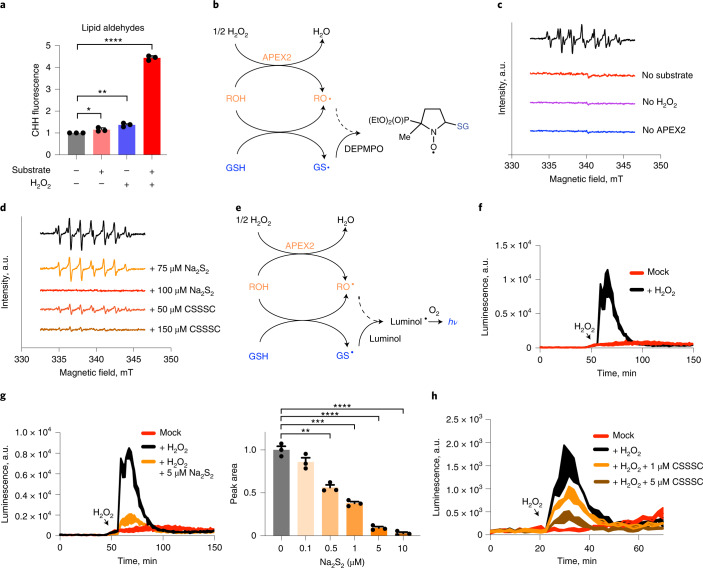
Exogenously supplied S^0^ eliminates intracellular radicals. **a**, Relative lipid aldehyde levels as measured by CHH staining in APEX2-expressing HeLa cells treated with substrate (HPI, 1 mM), H_2_O_2_ (100 µM, repeated every hour for 5 hours) or the combination of substrate and H_2_O_2_. *n* = 3. *P* = 0.0428, 0.0015 and 0.00001. **b**, Reaction scheme depicting APEX2-dependent generation of phenoxyl (RO•) and glutathionyl (GS•) radicals and their trapping with DEPMPO. **c**, ESR spectrum of HeLa cells incubated with DEPMPO (50 mM) in the presence of APEX2 expression, substrate (HPI, 1 mM) and H_2_O_2_ (1 mM) (upper trace). The lower traces show the spectra obtained when omitting either the substrate or H_2_O_2_ or in the absence of APEX2 expression. **d**, ESR spectrum of APEX2-expressing HeLa cells incubated with DEPMPO (50 mM) in the presence of the substrate (HPI, 1 mM) and H_2_O_2_ (1 mM) (upper trace). The lower traces show the influence of pre-treatment with either Na_2_S_2_ or CSSSC at the indicated concentrations. **e**, Reaction scheme depicting APEX2-dependent generation of phenoxyl (RO•) and glutathionyl (GS•) radicals and their further reaction with luminol to trigger light (hv) emission. **f**, Luminescence profiles recorded from APEX2-expressing HeLa cells in the presence of substrate (HPI, 250 µM) and luminol (250 µM), with or without the addition of H_2_O_2_ (50 µM). *n* = 3. **g**, Luminescence profiles recorded from APEX2-expressing HeLa cells in the presence of luminol (250 µM), substrate (HPI, 250 µM) and H_2_O_2_ (50 µM), with or without the persulfide donor Na_2_S_2_ (5 µM) (left panel). Titration of Na_2_S_2_ (0.1–10 µM) and quantitation of the area under the luminescence curve (right panel). Right panel: *n* = 3. *P* = 0.0012, 0.0002, 0.00004 and 0.00003. **h**, Luminescence profiles recorded from APEX2-expressing HeLa cells in the presence of luminol (250 µM), substrate (HPI, 250 µM) and H_2_O_2_ (50 µM), with or without the persulfide donor CSSSC (1 µM and 5 µM). *n* = 3. Data are presented as mean values. Error bars represent s.d. * *P* ≤ 0.05; ** *P* ≤ 0.01; *** *P* ≤ 0.001; and **** *P* ≤ 0.0001 based on a two-tailed unpaired *t*-test. a.u., arbitrary units. Source data

**Extended Data Fig. 5:**
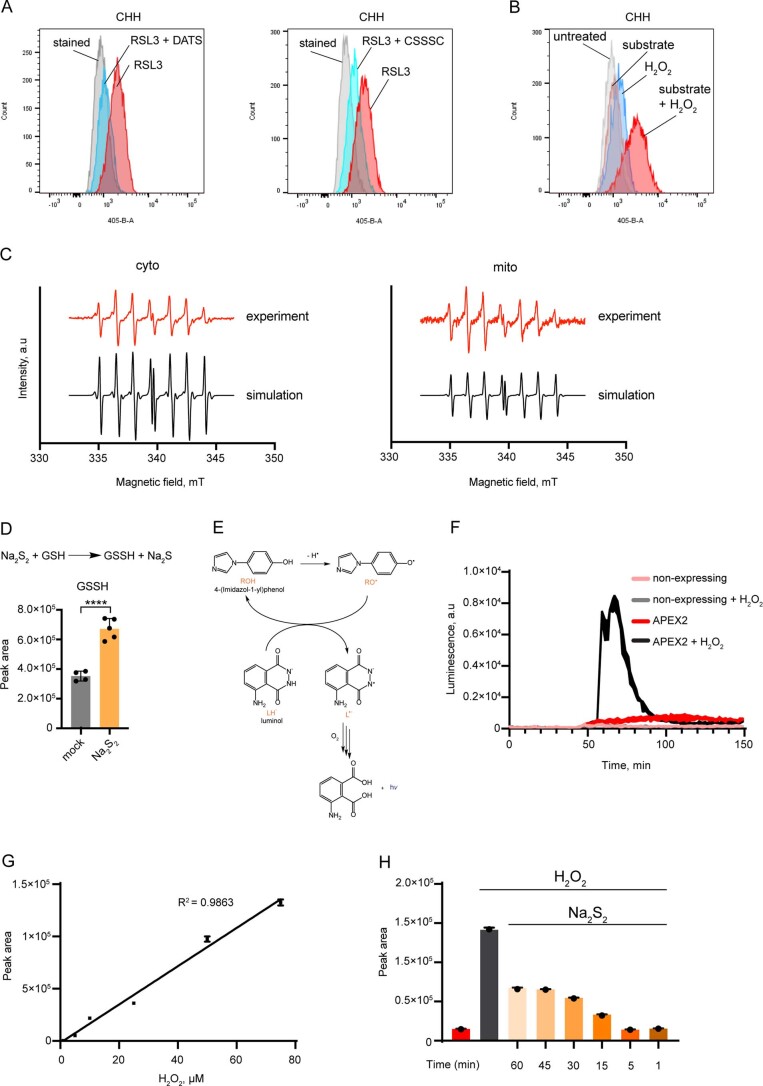
Exogenously supplied persulfide donors scavenge APEX2-generated intracellular radicals. (**A**) Flow cytometry of CHH-stained HeLa cells treated with RSL3 (5 µM) for 4 hours, in the presence or absence of DATS (100 µM) (left panel), and in the presence or absence of CSSSC (100 µM) (right panel). (**B**) Flow cytometry of CHH stained APEX2 expressing HeLa cells treated with substrate (HPI, 1 mM), H_2_O_2_ (100 µM, repeated hourly for 5 h) or with the combination of substrate and H_2_O_2_. (**C**) ESR spectra of DEPMPO-trapped radicals, recorded from HeLa cells expressing APEX2 in either the cytosol (upper left trace) or the mitochondrial matrix (upper right trace), after exposure to HPI (1 mM), H_2_O_2_ (1 mM) and DEPMPO (50 mM). Simulated ESR spectra of DEPMPO-trapped radicals (80% GS·, 20% C·) (lower traces), based on the following parameters: DEPMPO-SG·(80 %): g_iso_ = 2.00440, a_H1_ = 15.4 G, a_H2_ = 0.9 G, a_p_ = 45.9 G, a_NO_ = 14.1 G; DEPMPO-C·(20 %): g_iso_ = 2.00420, a_H1_ = 22.1 G, a_H2_ = 3 G, a_p_ = 46.1 G, a_NO_ = 14.8 G. Spectra were simulated with MATLAB R2021a using the EasySpin 5.2.33 package. ESR parameters were taken from^50^. (**D**) GSSH levels in HeLa cells measured by LC-MS after treatment with Na_2_S_2_ (50 µM) for 5 min. n = 4 (mock), n = 5 (Na_2_S_2_), p = 0.00007. (**E**) Reaction scheme explaining how APEX2-derived radicals generate luminescence in the presence of luminol. The APEX2 substrate 4-(Imidazol-1-yl)phenol (HPI) is oxidized by APEX2 to generate phenoxyl radicals, which further react with luminol to generate luminol radicals. The oxidation of luminol radicals by O_2_ leads to the emission of blue light. (**F**) Luminescence recorded from HeLa cells either expressing or not expressing APEX2, pre-treated for 20 min with luminol and substrate (HPI) (250 µM each), and then exposed to H_2_O_2_ (50 µM). n = 3. (**G**) Area under the luminescence curve obtained for different H_2_O_2_ concentrations, using the same conditions as in (F). (**H**) Area under the luminescence curve after incubating APEX2 expressing HeLa cells with Na_2_S_2_ (5 µM) for the indicated times, prior to the addition of H_2_O_2_ (50 µM). n = 3. Data are presented as mean values. Error bars represent SD, **** *P* ≤ 0.0001 based on a two-tailed unpaired *t*-test. Source data

We then asked if exogenously supplied membrane-permeable hydropersulfide donors could also protect cells against LPO. Indeed, addition of either diallyl tetrasulfide (DATS) or cysteine trisulfide (CSSSC) to the cell culture medium suppressed RSL3-induced LPO (Extended Data Fig. 5a). Having established that S^0^ contributes to preventing LPO and ferroptosis, we next set out to understand the underlying molecular mechanism. Because LPO is driven by radical chain reactions, we considered the possibility that S^0^ species act by quenching endogenous radicals. To investigate this possibility, we developed a chemogenetic system that allowed us to generate endogenous radicals in a controlled manner. To this end, we stably expressed the engineered heme peroxidase APEX2 (ref. ^23^) in either the cytosol or mitochondria of HeLa cells. APEX2 uses H_2_O_2_ to generate phenoxyl radicals from phenolic substrates. Co-treatment of APEX2-expressing cells with H_2_O_2_ and the phenolic peroxidase substrate HPI (1-(p-hydroxyphenyl) imidazole) enhanced LPO in a synergistic manner (Fig. 3a and Extended Data Fig. 5b). To directly measure the formation of radicals in this system, we incubated cells with the membrane-permeable spin probe DEPMPO and subjected them to electron spin resonance (ESR) spectroscopy. We expected the resulting phenoxyl radicals to be largely reduced by GSH, in turn forming glutathionyl radicals (GS•), which should be trapped as characteristic DEPMPO adducts (Fig. 3b). The observed ESR signal was strictly dependent on the combined presence of APEX2, H_2_O_2_ and HPI, confirming the specificity of the system and the absence of background activities (Fig. 3c). The shape of the ESR spectrum indicated formation of mainly GS• by cytosolic or mitochondrial APEX2 (Extended Data Fig. 5c). Pre-treatment of cells with membrane-permeable inorganic or organic polysulfides, confirmed to generate endogenous GSSH (Extended Data Fig. 5d), suppressed the ESR signal in a concentration-dependent manner (Fig. 3d). To further consolidate these findings, we aimed to follow radical formation and elimination in real time. To this end, we used luminol instead of the spin trap (Fig. 3e). Radicals generated by APEX2 are expected to oxidize luminol to luminol radicals, which further react with molecular oxygen to emit blue light (Extended Data Fig. 5e). As expected, the addition of H_2_O_2_ to cells pre-incubated with the phenolic substrate and luminol induced transient luminescence (Fig. 3f) in a strictly APEX2-dependent manner (Extended Data Fig. 5f) and proportional to the amount of H_2_O_2_ added (Extended Data Fig. 5g). We then found that pre-treatment of cells with a membrane-permeable persulfide donor (Na_2_S_2_) suppressed H_2_O_2_-induced luminescence in a concentration-dependent and time-dependent manner (Fig. 3g and Extended Data Fig. 5h). Similar effects were recapitulated with the organic persulfide donor CSSSC (Fig. 3h). In sum, we found that exogenously supplied hydropersulfide donors lower endogenous radical levels.

### Endogenously produced S^0^ eliminates intracellular radicals

**Fig. 4:**
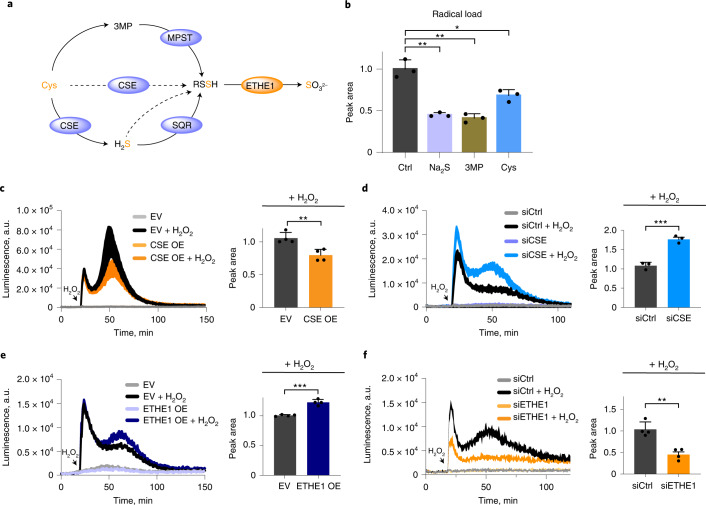
Endogenously produced S^0^ eliminates intracellular radicals. **a**, Selected enzymatic pathways of hydropersulfide (RSSH) formation and elimination. CSE drives RSSH formation primarily through production of H_2_S, which is oxidized to RSSH by SQR. MPST provides an H_2_S-independent route to RSSH. Hydropersulfides are degraded by the persulfide dioxygenase ETHE1, which oxidizes S^0^ to sulfite (SO_3_^2−^). **b**, Influence of Na_2_S (5 µM, substrate for SQR), 3MP (100 µM, substrate for MPST) or Cys (1 mM, substrate for CSE) on the radical load of APEX2-expressing HeLa cells, added 5 minutes before triggering radical generation with luminol, substrate and H_2_O_2_ (50 µM). *n* = 3. *P* = 0.0011, 0.0011 and 0.0136. **c**–**f**, Influence of CSE overexpression (**c**), CSE depletion (**d**), ETHE1 overexpression (**e**) and ETHE1 depletion (**f**) on the luminescence profiles recorded from APEX2-expressing HeLa cells (left panels). Cells were incubated with luminol and substrate (250 µM each), and radical generation was triggered with H_2_O_2_ (50 µM). Normalized AUC (right panels). EV, empty vector. *n* = 4. *P* = 0.0056 (**c**). *n* = 3. *P* = 0.0009 (**d**). *n* = 4. *P* = 0.0001 (**e**). *n* = 4. *P* = 0.0016 (**f**). Data are presented as mean values. Error bars represent s.d. * *P* ≤ 0.05; ** *P* ≤ 0.01; *** *P* ≤ 0.001; and **** *P* ≤ 0.0001 based on a two-tailed unpaired *t*-test. a.u., arbitrary units; OE, overexpressing. Source data

**Extended Data Fig. 6:**
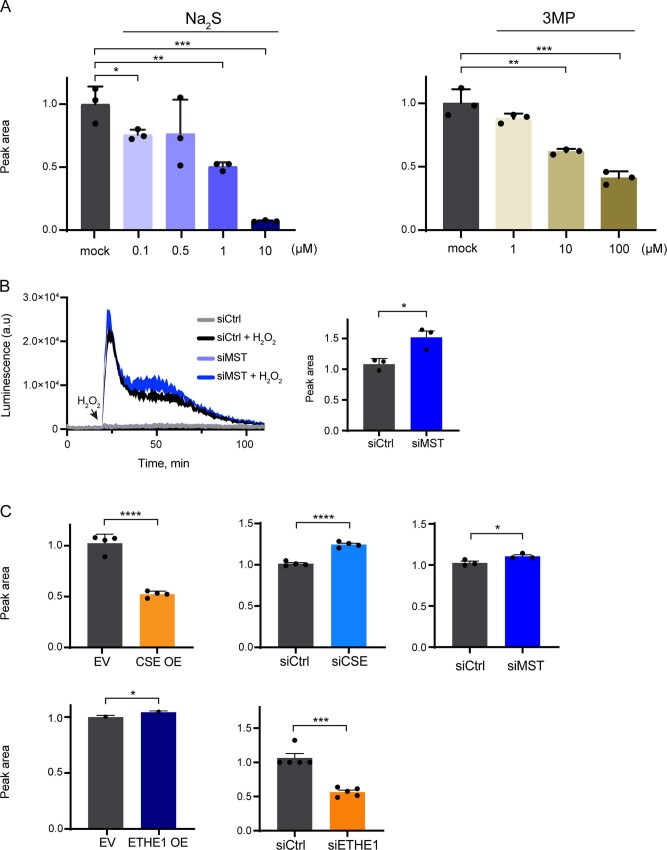
Endogenously generated persulfides scavenge APEX2-generated intracellular radicals. (**A**) Area under the luminescence curve obtained after pretreating APEX2 expressing HeLa cells with the indicated concentrations of Na_2_S (left panel) or 3MP (right panel) for 5 min before the addition of H_2_O_2_ (50 µM). n = 3. p = 0.0451. p = 0.0040. p = 0.0003. (**B**) Influence of MPST depletion on the luminescence recorded from APEX2-expressing HeLa cells after addition of H_2_O_2_ (50 µM) (left panel). Corresponding quantitation of the area under the luminescence curve (right panel). n = 3. p = 0.0198. (**C**) Influence of CSE overexpression (upper left panel), CSE depletion (upper middle panel), MPST depletion (upper right panel), ETHE1 overexpression (lower left panel), and ETHE1 depletion (lower right panel) on the area under the luminescence curve measured in HeLa cells expressing APEX2 in the mitochondrial matrix. n = 4. p = 0.00004. p = 0.00004. (CSE OE, siCSE); n = 3. p = 0.0435 (siMST); n = 4. p = 0.0474 (ETHE OE); n = 5. p = 0.0001 (siETHE1). Data are presented as mean values. Error bars represent SD, * *P* ≤ 0.05; ** *P* ≤ 0.01; *** *P* ≤ 0.001; **** *P* ≤ 0.0001 based on a two-tailed unpaired *t*-test. Source data

Next, we asked if endogenously produced persulfides are able to eliminate intracellular radicals. To this end, we manipulated the enzymes involved in S^0^ metabolism (Fig. 4a) in APEX2-expressing cells. The exogenous addition of substrates for CSE (Cys), MPST (3MP) or SQR (H_2_S) lowered endogenous radical load, in line with the notion that enzymatically generated S^0^ species contribute to radical scavenging (Fig. 4b and Extended Data Fig. 6a). Indeed, overexpression of CSE decreased radical load (Fig. 4c), whereas its depletion led to an increase (Fig. 4d). Conversely, overexpression of ETHE1 increased radical load (Fig. 4e), whereas its depletion resulted in a decrease (Fig. 4f). Depletion of MPST also increased radical load, albeit to a lesser extent than depletion of CSE (Extended Data Fig. 6b). Similar results were obtained when APEX2 was expressed in the mitochondrial matrix instead of the cytosol (Extended Data Fig. 6c).

### Hydropersulfides are potent radical scavengers

**Fig. 5:**
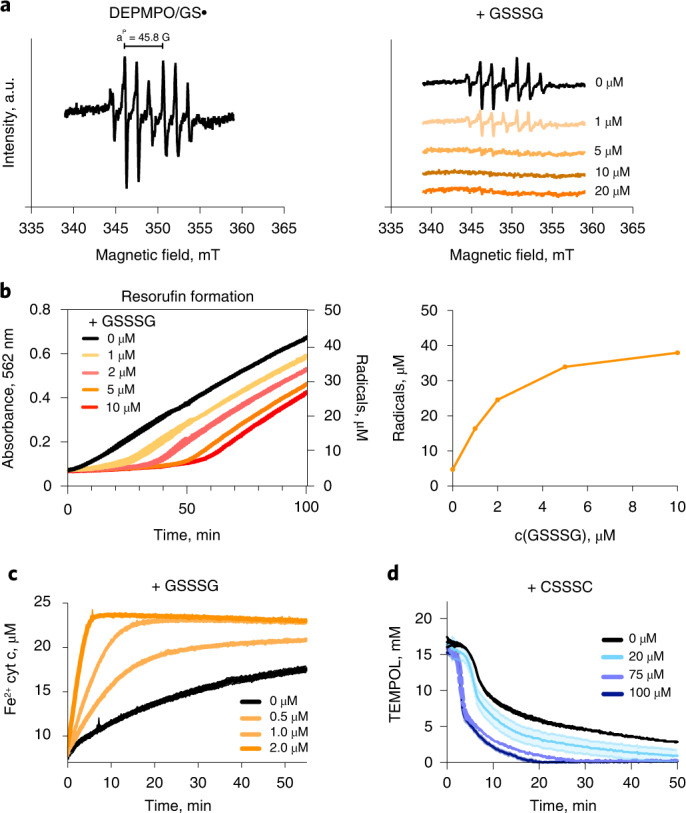
Persulfides are superior radical scavengers. **a**, In vitro ESR spectrum of GS• radicals spin trapped in a solution containing DEPMPO (10 mM), GSH (1 mM), APEX2 (1 µM) and H_2_O_2_ (100 µM) (left panel). Corresponding ESR spectra obtained by the addition of the indicated concentrations of the persulfide donor GSSSG (right panel). **b**, Resorufin formation recorded from a mixture of APEX2 (2 µM), Amplex Red (100 µM), GSH (1 mM) and increasing concentrations of GSSSG (left panel). Using an H_2_O_2_ calibration curve, the observed inhibition time allows the calculation of the amount of radicals eliminated in the presence of GSSSG (right panel). *n* = 2. **c**, Reduction of ferric cytochrome c (cyt c, 24 µM) measured by absorbance at 550 nm in the presence of GSH (1 mM) and the indicated concentrations of GSSSG. *n* = 2. **d**, Reduction of TEMPOL (18 mM) measured by absorbance at 430 nm in the presence of GSH (20 mM) and the indicated concentrations of CSSSC. *n* = 2. a.u., arbitrary units. Source data

**Extended Data Fig. 7:**
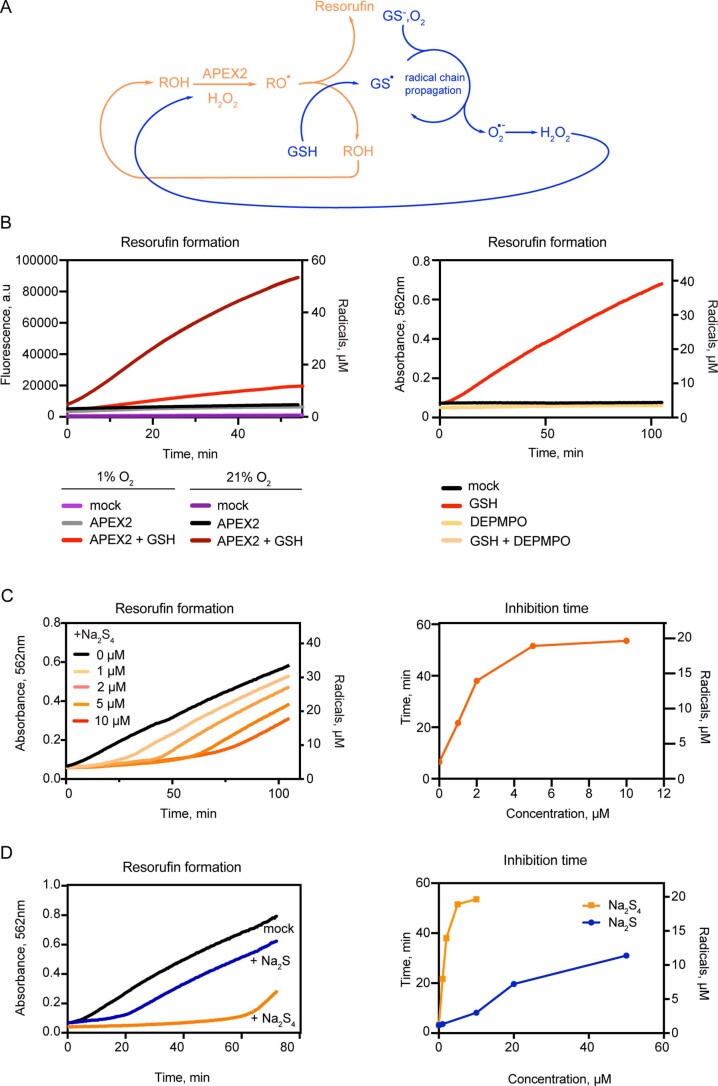
Persulfides are superior radical scavengers. (**A**) Scheme explaining the autoxidation of Amplex Red (ROH) in the presence of GSH, APEX2 and O_2_. APEX2-generated phenoxyl radicals (RO·) react with GSH to form glutathionyl radicals (GS·). These react further with GSH and O_2_ to produce superoxide radicals (O2^.-^), which dismutate to yield H_2_O_2_, in turn driving APEX2 activity. Phenoxyl radicals of Amplex Red disproportionate to form Amplex Red and resorufin. (**B**) Influence of oxygen partial pressure (1% vs. 21%) on resorufin formation in the presence of APEX2 (2 µM) and Amplex Red (100 µM), with or without GSH (1 mM) (left panel). Influence of DEPMPO (20 mM) on resorufin formation in the presence of APEX2 (2 µM) and Amplex Red (100 µM), with or without GSH (1 mM) (right panel). n = 2. (**C**) Influence of increasing concentrations of Na_2_S_4_ on resorufin formation in the presence of APEX2 (2 µM), Amplex Red (100 µM), GSH (1 mM) and 21% O_2_ (left panel). The relationship between Na_2_S_2_ concentration and inhibition time (left axis) allows quantification of eliminated radicals (right axis), using a calibration curve (right panel). n = 2. (**D**) Influence of Na_2_S (10 µM) on resorufin formation in the presence of APEX2 (2 µM), Amplex Red (100 µM), GSH (1 mM) and 21% O_2_. The influence of Na_2_S_4_ (10 µM) is shown for comparison (left panel). The relationship between Na_2_S concentration and inhibition time, shown in comparison to Na_2_S_4_ (right panel). n = 2. Data are presented as mean values. Source data

**Extended Data Fig. 8:**
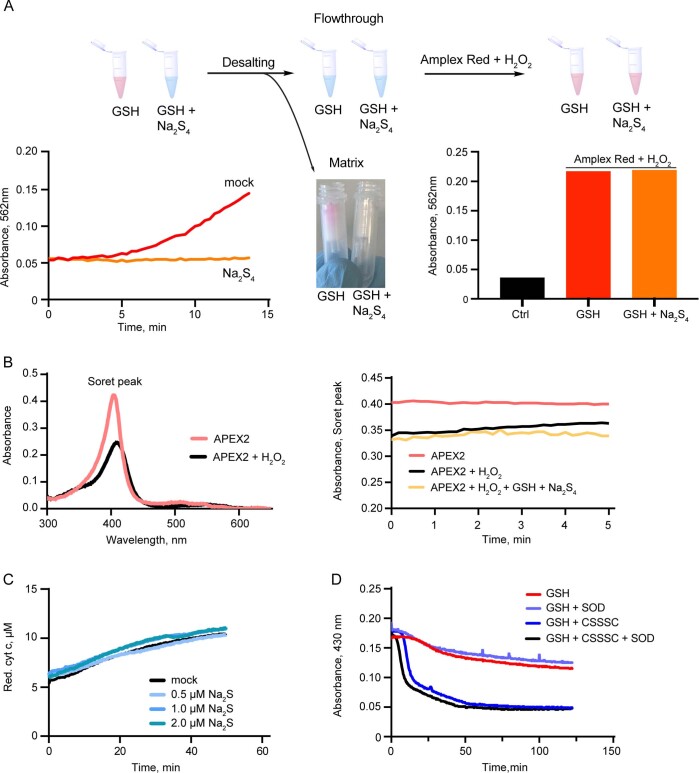
Persulfides do not inhibit APEX2 or cytochrome c. (**A**) APEX2 is not inactivated by polysulfides. APEX2 (2 µM) was incubated with Amplex Red (100 µM), GSH (1 mM), plus/minus Na_2_S_4_ (10 µM), for 15 min, while recording resorufin formation (left panel). Next, APEX2 was separated from low molecular weight compounds by gel filtration. The photo shows resorufin remaining in the gel filtration matrix (middle panel). When desalted APEX2 was again incubated with Amplex Red (100 µM) and H_2_O_2_ (100 µM) its activity was unaffected by prior exposure to Na_2_S_4_. For measurement, samples were diluted 20-fold (right panels). (**B**) Absorbance spectra of APEX2 (5 µM) in the presence or absence of H_2_O_2_ (10 µM) (left panel). Recording of the APEX2 Soret peak (absorbance at 406 nm) from a mixture of APEX2 (5 µM) and H_2_O_2_ (10 µM), to which GSH (1 mM) and Na_2_S_4_ (10 µM) were added (right panel). (**C**) Reduction of cyt c (24 µM) over time, in the presence of GSH (1 mM) and the indicated concentrations of Na_2_S, measured by absorbance at 550 nm. n = 2. (**D**) Reduction of TEMPOL (18 mM) over time, in the presence of GSH (20 mM), plus/minus CSSSC (100 µM) and plus/minus SOD (10 U/mL), as measured by absorbance at 430 nm. n = 2. Data are presented as mean values. Source data

To obtain further mechanistic insight, we studied the GSSH-generating GSSSG/GSH system and its interaction with radicals in vitro. We used recombinant APEX2 in combination with Amplex Red (resazurin) and H_2_O_2_ to generate radicals in the presence of millimolar amounts of GSH. Adding the DEPMPO spin trap, the ESR spectrum confirmed formation of the glutathione adduct (Fig. 5a, left panel). Spiking the system with low micromolar concentrations of GSSSG (generating corresponding amounts of GSSH) suppressed the ESR signal (Fig. 5a, right panel). We then performed the same experiment without adding H_2_O_2_ or the spin trap, relying on radical-dependent and O_2_-dependent H_2_O_2_ formation, similarly to Amplex Red autoxidation to resorufin in the presence of horseradish peroxidase (HRP)^24^ (Extended Data Fig. 7a). As expected, resorufin formation was inhibited under hypoxic conditions or in the presence of the spin trap (Extended Data Fig. 7b). The addition of increasing micromolar amounts of GSSSG inhibited resorufin formation for increasing periods of time (Fig. 5b, left panel). Based on resorufin absorbance and the observed inhibition time, the amount of scavenged radicals was calculated, indicating that low amounts of GSSSG scavenge >10 times the amount of radicals (Fig. 5b, right panel). Addition of the inorganic persulfide donor Na_2_S_4_ instead of GSSSG achieved similar results (Extended Data Fig. 7c). Another experiment confirmed that H_2_S (as potentially formed by GSH-mediated GSSH reduction) is unlikely to be involved in radical scavenging: Na_2_S inhibited resorufin formation only weakly (Extended Data Fig. 7d), and this inhibition is likely due to polysulfide contaminations typically accompanying commercial Na_2_S preparations^25^. Additional experiments confirmed that persulfides and polysulfides do not block APEX2 activity (Extended Data Fig. 8a) and that neither GSH nor GSSH can reduce compound I of APEX2 (Extended Data Fig. 8b). Similarly to the resazurin/resorufin system, GSSSG accelerated GSH-dependent 1e^−^ reduction of cytochrome c (cyt c) at sub-stoichiometric concentrations (Fig. 5c), whereas H_2_S did not facilitate any cyt c reduction (Extended Data Fig. 8c). Likewise, addition of sub-stoichiometric amounts of CSSSC accelerated the reduction of TEMPOL radicals by GSH (Fig. 5d), without involvement of superoxide (Extended Data Fig. 8d).

### GSSH catalyzes GSH-dependent radical reduction

**Fig. 6:**
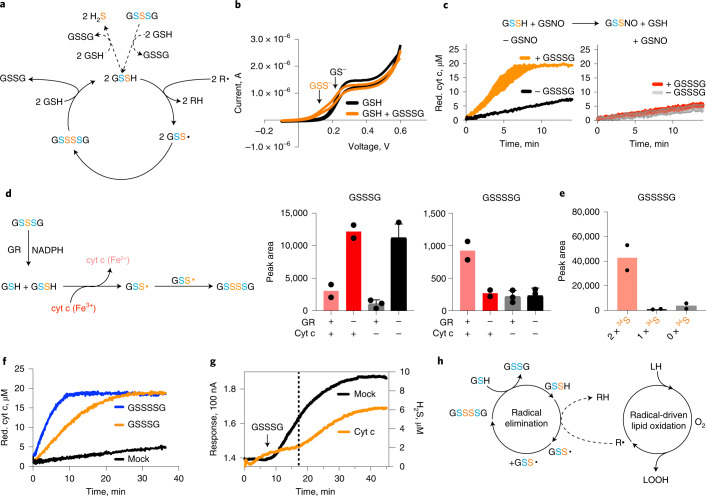
GSSH catalyzes GSH-dependent radical reduction. **a**, Proposed autocatalytic cycle of GSSH-mediated radical elimination. GSSH reduces radicals (R•), in turn forming perthiyl radicals (GSS•). These recombine to form GSSSSG, which is then reduced by GSH to regenerate GSSH. Overall, the cycle couples glutathione oxidation to radical reduction. Potential supply and decay pathways for GSSH are indicated by dotted lines. For example, GSSH can be formed from the persulfide donor GSSSG. In the absence of radicals, GSSH is slowly reduced by GSH, releasing H_2_S. **b**, Cyclic voltammetry of a GSH solution with or without the addition of GSSSG. **c**, Influence of blocking GSSH with the NO donor GSNO. Reduction of ferric cyt c (20 µM) was measured in the presence of GSH (1 mM) and GSSSG (1 µM), and in the presence or absence of GSNO (1 mM). *n* = 2. **d**, Reduction of ferric cyt c triggers the formation of GSSSSG. Using GR (1 U ml^−1^) and NADPH (400 µM), GSSSG (400 µM) is rapidly reduced to GSSH. GSSSSG is specifically formed in the presence of ferric cyt c, thus implicating the formation and recombination of perthiyl radicals. GSSSG and GSSSSG levels were measured by LC–MS (right panel). *n* = 2 (first two bars) and *n* = 3 (remaining bars). **e**, Conversion of singly labeled glutathione trisulfide (GS^34^SSG) to doubly labeled glutathione tetrasulfide (GS^34^S^34^SSG) by the GR/NADPH/cyt c system, as measured by LC–MS. *n* = 2. **f**, Acceleration of cyt c reduction by GSSSSG. The reduction of ferric cyt c (20 µM) was measured in the presence of GSH (1 mM) and either GSSSG or GSSSSG (1 µM). *n* = 2. **g**, H_2_S release from the mixture of ferric cyt c (50 µM), GSH (1 mM) and GSSSG (10 µM), as measured with an H_2_S-selective electrode. The vertical dashed line indicates the timepoint of complete cyt c reduction. *n* = 2. **h**, Scheme summarizing the radical chain braking effect of persulfides. Initiating and/or propagating radials (R•) are rapidly reduced by hydropersulfides (GSSH). The resulting perthiyl radicals (GSS•) do not participate in radical chain propagation but, instead, rapidly self-recombine to form GSSSSG, thus permanently removing unpaired electrons from the system. Reduction of GSSSSG regenerates GSSH. Data are presented as mean values. Error bars represent s.d. Source data

**Extended Data Fig. 9:**
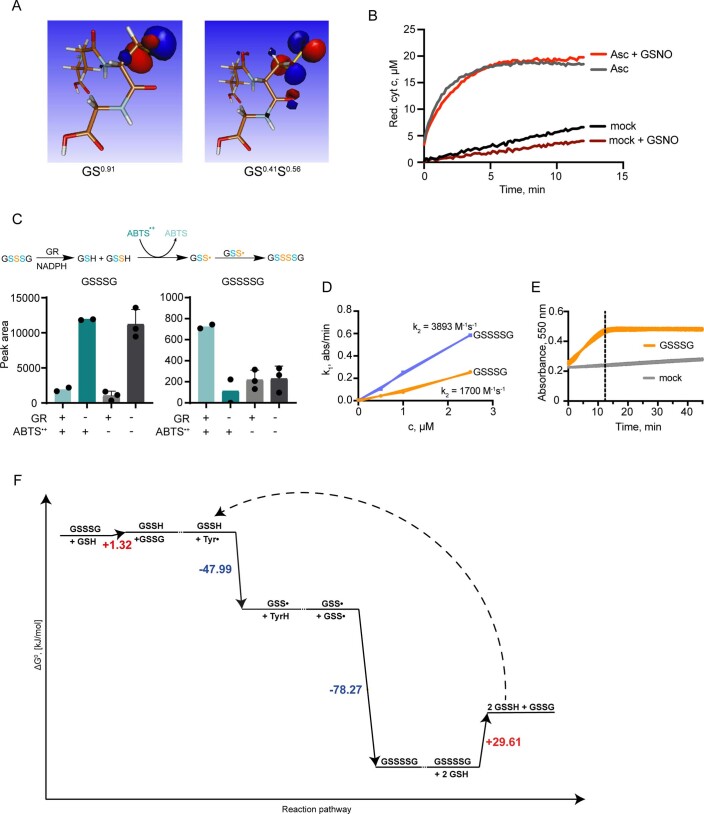
GSSH catalyzes the reduction of radicals by GSH. (**A**) Visualization of the singly occupied molecular orbital in GS· (left panel) and GSS· (right panel). The corresponding spin radical densities are given below the panels. (**B**) Reduction of cyt c (20 µM) over time, in the presence of GSH (1 mM), plus/minus ascorbate (Asc, 0.5 mM), plus/minus S-nitrosoglutathione (GSNO, 1 mM), measured by absorbance at 550 nm. n = 2. (**C**) In the presence of radicals, GSSH converts into GSSSSG. We used GR (1 U/mL) plus NADPH (400 µM) to rapidly convert GSSSG (400 µM) into GSSH. By reducing ABTS radicals (400 µM), GSSH converts into perthiyl radicals (GSS·) which in turn recombine to yield GSSSSG (upper panel). Measurement of GSSSG (lower left panel) and GSSSSG levels (lower right panel) by LC-MS. n = 2 (first two bars). n = 3 (remaining bars). (**D**) Kinetics of GSH-mediated cyt c reduction in the presence of either GSSSG or GSSSSG. n = 2. (**E**) Reduction of cyt c (50 µM) in the presence of GSH (1 mM) plus/minus GSSSG (10 µM), measured by absorbance at 550 nm. The vertical dashed line indicates the time point of complete cyt c reduction. n = 2. **(F)** QM calculation of standard state Gibbs free energies (ΔG^0^) for all reactions in the proposed reaction cycle, shown here for the reduction of tyrosyl radicals (Tyr·). Of note, calculation of ∆G values on the basis of estimated intracellular concentrations would result in negative ∆G values for GSH-mediated reduction steps (first and last steps). Data are presented as mean values. Error bars represent SD. Source data

Having observed that radicals are efficiently scavenged in the presence of sub-stoichiometric amounts of persulfides, we considered the possibility that persulfides couple radical reduction to glutathione oxidation in a self-regenerating manner. We, therefore, considered an autocatalytic cycle in which GSSH reduces radicals to form perthiyl radicals (GSS•), which recombine to form glutathione tetrasulfide (GSSSSG), which is then reduced by GSH to regenerate GSSH (Fig. 6a). Using cyclic voltammetry, we confirmed that GSSH has a much lower reduction potential than GSH (Fig. 6b). In line with this result, quantum mechanical (QM) calculations showed the unpaired electron of GSS• to be de-localized between the two sulfur atoms and confirmed the higher stability of GSS• relative to GS• (Extended Data Fig. 9a). The nitric oxide (NO) donor GSNO (facilitating S-nitrosylation of GSSH) completely abolished GSSSG-dependent cyt c reduction (Fig. 6c), without inhibiting cyt c (Extended Data Fig. 9b). We furthermore confirmed by mass spectrometry (MS) that the reaction of GSSH with either ferric cyt c or ABTS radicals indeed generates GSSSSG (Fig. 6d and Extended Data Fig. 9c), implying the recombination of GSS• radicals. When we used GS^34^SSG to generate singly labeled GS^34^SH, we observed the generation of doubly labeled GS^34^S^34^SSG, again implying perthiyl radical recombination (Fig. 6e and Supplementary Fig. 2). As predicted by the model, the catalytic cycle can also be initiated by providing GSSSSG instead of GSSSG (Fig. 6f and Extended Data Fig. 9d). Furthermore, the model predicts competition between radical-dependent and GSH-dependent GSSH reduction. Indeed, we found that H_2_S formation is suppressed until cyt c is completely reduced (Fig. 6g and Extended Data Fig. 9e). Finally, QM calculations support the notion that the proposed persulfide cycle is thermodynamically favorable (Extended Data Fig. 9f). In conclusion, several independent lines of evidence support the concept of an autocatalytic persulfide cycle driven by radicals.

## Discussion

In this study, we explored connections among Cys availability, hydropersulfide levels, free radical levels, LPO and ferroptosis sensitivity. Our starting point was the observation that cells primed to undergo ferroptosis increase their endogenous S^0^ levels. This finding prompted us to consider the possibility that S^0^ species (typically persulfides and polysulfides) somehow act against ferroptosis and that their upregulation is an adaptive response to pro-ferroptotic conditions. This hypothesis seemed reasonable, as S^0^ species have previously been observed to be upregulated under oxidative stress conditions^26^ and to exert cytoprotective effects^16,27^.

Cys is known to be the ultimate source of S^0^ in persulfides and polysulfides^14,21^. Hence, we reasoned that Cys uptake may counteract ferroptosis not only through the well-established Cys–GSH–GPX4 axis but also by enabling biosynthesis of S^0^ species. Indeed, we found that increased intracellular Cys availability can suppress lipid oxidation (and, hence, ferroptosis) independently of the canonical GPX4 system.

If persulfides naturally play a role in limiting LPO, the expression levels of enzymes involved in persulfide generation and turnover can be expected to influence lipid oxidation levels and ferroptosis sensitivity. To this end, we mainly focused on the manipulation of two enzymes. One enzyme, CSE, supports the biosynthesis of persulfides, primarily by generating H_2_S from Cys, but potentially also by generating cysteine persulfide from cystine^14^. The other enzyme, ETHE1, degrades persulfides (GSSH + O_2_ + H_2_O → GSH + SO_3_^2−^ + 2 H^+^)^14^. We found the two enzymes to have opposing effects on membrane integrity (Fig. 2c) and LPO (Fig. 2d,e). The pro-ferroptotic effect of CSE depletion aligns with a recent study reporting that depletion of cystathionine β-synthase (CBS), another enzyme capable of supporting persulfide biosynthesis, promotes ferroptosis in breast cancer cells. CBS depletion was observed to diminish persulfide levels, but not Cys or GSH levels, thus implicating its non-canonical H_2_S/S^0^-generating function in ferroptosis protection^28^. A potential caveat in interpreting CSE or CBS effects is that SQR-dependent oxidation of H_2_S (as generated by CSE or CBS) not only yields GSSH but also feeds electrons into the mitochondrial CoQ_10_ pool. However, recent work on the CoQ oxidoreductase FSP1 has provided some clear indications that it is the extra-mitochondrial CoQ_10_ pool at the plasma membrane that is relevant for ferroptosis protection^7,8^. Assessing the relative significance of individual S^0^-generating enzymes (including CSE, CBS, SQR, MPST and CARS) in ferroptosis, depending on cell type and metabolic context, will require further analysis.

A central finding of our study is that exogenously supplied or endogenously produced persulfides facilitate the rapid removal of endogenously produced free radicals. We used the engineered heme peroxidase APEX2 as a chemogenetic tool to generate free radicals and drive lipid oxidation. Spin trapping in living cells revealed that mostly glutathionyl radicals, previously shown to facilitate LPO^29^, were generated in this setting. Using luminescence-based real-time monitoring of radical levels, we observed that low concentrations of exogenously supplied membrane-permeable persulfide donors (CSSSC, Na_2_S_2_) accelerate endogenous radical removal (Fig. 3). Manipulating CSE and ETHE1 levels in both directions, we found a consistent pattern in that the two enzymes mirrored each other: silencing CSE yielded the same effect (slower radical removal) as overexpressing ETHE1, and overexpressing CSE yielded the same effect (faster radical removal) as silencing ETHE1 (Fig. 4). In addition, we depleted another persulfide-generating enzyme, MPST, and also observed slower radical removal.

These observations raised the question of how hydropersulfides actually eliminate radicals. To approach this question, we studied persulfide–radical interactions in vitro. To our surprise, we observed non-stoichiometric effects—that is, more radicals were eliminated than persulfides were added to the system (Fig. 5). This led us to consider a three-step autocatalytic cycle in which persulfides are regenerated (Fig. 6a). We further realized that a catalytic role for persulfides in 1-electron reactions had already been proposed more than 50 years ago. In 1971, it was reported that, in the presence of excess GSH, one molecule of CSSSC led to the reduction of at least 25 molecules of cytochrome c, implicating hydropersulfides as catalysts^30^.

The first step of the proposed cycle is the reduction of radicals by hydropersulfides. Our in vitro experiments strongly suggest that hydropersulfides are highly efficient in directly reducing radicals. This conclusion is in line with and supported by recent findings. Hydropersulfides were found to be excellent H-atom donors to radicals, including alkyl, alkoxyl, peroxyl and thiyl radicals^31^. Their inherent reactivity to chain-carrying peroxyl radicals is four orders of magnitude greater than for thiols and essentially the same as that of α-tocopherol. However, hydropersulfides are superior to α-tocopherol owing to their low H-bond acidity, a unique attribute of hydropersulfides, which makes their reactivity largely insensitive to changes in the surrounding medium^20^. It may be argued that radical scavenging by hydropersulfides is nevertheless limited by their inherent instability, as they are subject to thiol-mediated reduction in the presence of millimolar amounts of GSH (GSSH + GSH → GSSG + H_2_S). However, hydropersulfides are continuously re-synthesized by enzymatic activity and available at micromolar steady-state concentrations^15^. When radicals appear, their reaction with persulfides should outcompete GSH-dependent persulfide reduction, which is a comparably slow process. Indeed, we observed in our in vitro experiments that the decay of persulfides to H_2_S was strongly suppressed in the presence of radicals. Hence, in the presence of radicals, other persulfide-degrading processes likely become kinetically irrelevant. The direct reduction of radicals by hydropersulfides leads to the formation of perthiyl radicals. These have unique properties. They are extraordinarily stable in terms of reactivity toward other molecules. They have a very low tendency to pass on oxidizing equivalents by extracting H-atoms from other molecules^31^. Nevertheless, perthiyl radicals have a fleeting (almost diffusion-limited) existence, as they have a strong tendency to recombine with each other. In other words, perthiyl radicals persist until they encounter another perthiyl radical with which they recombine to form tetrasulfides^18^. Perthiyl radicals are usually not observed to react with other radicals or with O_2_ (refs. ^32,33^), probably because these reactions are outcompeted by rapid self-recombination.

The second step in the cycle is, thus, the recombination of perthiyl radicals at a rate close to the diffusion limit^18^. Perthiyl radicals seem to be unique among all other biological radicals by almost exclusively engaging in self-recombination. Hence, the formation and recombination of perthiyl radicals efficiently terminates radical chains^18^. In contrast, the thiyl radical propagates radical chain reactions^34,35^, and even the long-lived ɑ-tocopherol radical does so^36^. The failure to directly detect GSS• by us and others^31,32^ is in line with their stability toward other molecules (including spin traps) on the one hand and their rapid disappearance (through self-recombination) on the other. Accordingly, our evidence for their formation is indirect. However, we detected the product of recombination, GSSSSG, and confirmed that it was formed in a radical-dependent manner. Furthermore, isotopic labeling of GSSH led to the formation of doubly labeled GSSSSG, in support of perthiyl recombination (Fig. 6e).

The third step in the cycle is the reduction of GSSSSG to regenerate GSSH. In the presence of millimolar amounts of GSH, GSSSSG is subject to reduction. GSSSSG may, in principle, regenerate two GSSH molecules (net reaction: GSSSSG + 2 GSH → GSSG + 2 GSSH). It is conceivable that GSSSSG reduction can be catalyzed by enzymes. Glutathione reductase (GR) is known to accept GSSSG as a substrate^37^ and may also act on GSSSSG. Additionally, GSSSSG may also act as a radical scavenger by undergoing replacement reactions with radicals (GSSSSG + R• → GSSR + GSS•)^38,39^, thus regenerating perthiyl radicals, which, again, recombine to yield GSSSSG. Irrespective of the detailed steps, the recycling of S^0^ in a persulfide cycle may explain why low micromolar amounts of GSSH can be efficient radical scavengers in the presence of a large excess of GSH (Fig. 6h).

Another key question is how small molecule persulfides, such as GSSH, which are mostly hydrophilic, can intercept radical chain reactions taking place within the hydrophobic environment of a lipid bilayer. We can see several possibilities, which are not mutually exclusive. First, water-soluble hydropersulfides may reduce initiator radicals formed within the aqueous phase—for example, thiyl radicals (GS•), which are known to initiate LPO^29,40^. Intracellular APEX2 mostly generates GS• (Extended Data Fig. 3c) and, at the same time, drives lipid oxidation (Fig. 3a). In the same system, we observe the elimination of GS• in the presence of S^0^ species (Fig. 3d,g). These findings are compatible with the idea that persulfides intercept initiator radicals before they can start new chain reactions. Second, water-soluble hydropersulfides may reduce α-tocopherol and/or ubiquinone (CoQ_10_) radicals, which are accessible for interactions on the membrane surface. The coupling of lipophilic and hydrophilic radical scavengers is a well-known principle in redox biology. Tocopherols reduce lipid radicals within the membrane, and the resulting tocopherol radicals are then directly re-reduced by ascorbate^41^. Another example is lipid radical scavenging by CoQ_10_, which can also couple to water-soluble reductants. Indeed, the oxidoreductase FSP1 protects against LPO and ferroptosis by transferring electrons from NAD(P)H to oxidized CoQ_10_ (refs. ^7,8^). Thus, it seems plausible that hydropersulfides such as GSSH have access and the ability to reduce tocopherol and/or ubiquinone radicals. It has been reported that GSH can couple to the tocopherol system by directly reducing the tocopherol radical^41^. Given the 100-mV lower redox potential of GSSH relative to GSH (Fig. 6b), GSSH would be a much better reductant for tocopherol radicals. If GSH can interact with tocopherol on the membrane surface, the same should be true for GSSH. Third, water-soluble hydropersulfides may directly reduce chain-propagating lipid radicals (LOO•/LO•) if the oxidized fatty acid chain swings out to the membrane surface, due to its increased polarity, as suggested previously^42^. Fourth, there is also the possibility that hydrophilic persulfides/polysulfides give rise to lipophilic persulfide species. However, it is currently unknown if lipophilic persulfides are produced inside mammalian cells.

Taken together, our results support the notion that hydropersulfides provide protection against free radicals and associated chain reactions, as they react rapidly and in a potentially self-regenerating manner. Notably, the hydropersulfide/perthiyl system appears to be distinguished by an exceptional combination of chemical properties. By avidly reducing radicals, hydropersulfides essentially act as a sink for unpaired electrons. The resulting perthiyl radicals are resonance stabilized and are, therefore, unlikely to pass on the unpaired electron status to other molecules. Instead, they have a strong tendency to pair their unpaired electrons among each other (that is, by dimerization), thus eliminating unpaired electrons in the most direct way. Given its simplicity, the hydropersulfide/perthiyl system may represent an evolutionary ancient cellular radical removal system.

## Methods

### Chemicals and enzymes

3-aminophthalhydrazide (luminol), 1-(p-hydroxyphenyl)imidazole (HPI), disodium sulfide (Na_2_S), erastin, RSL3, cumene hydroperoxide (CHP), liproxstatin-1, 7-(diethylamino)coumarin-3-carbohydrazide (CHH), 4-hydroxytamoxifen (Tam), monobromobimane (MBB), cysteine (Cys), 3-mercaptopyruvate (3MP), cytochrome c (cyt c), glutathione reductase (GR), 2,2′-azino-bis(3-ethylbenzothiazoline-6-sulfonic acid) (ABTS), S-nitrosoglutathione (GSNO), ferrocene, 4-hydroxy-2,2,6,6-tetramethylpiperidin-1-oxyl (TEMPOL) and isopropyl β-d-1-thiogalactopyranoside (IPTG) were obtained from Sigma-Aldrich. BODIPY C11, diallyl tetrasulfide (DATS) and Amplex Red were obtained from Thermo Fisher Scientific. DEPMPO was obtained from Enzo. H_2_O_2_ was obtained from Roth. GSH and 5-aminolevulinic acid hydrochloride were obtained from Merck. Sodium disulfide (Na_2_S_2_), sodium tetrasulfide (Na_2_S_4_) and 3′,6′-di(O-thiosalicyl)fluorescein (SSP4) were obtained from Dojindo. The synthesis of GSSSG, GS^34^SSG and GSSSSG was performed as described previously^43^. CSSSC was synthesized as described below.

### Plasmids

The open reading frame (ORF) encoding APEX2 was obtained from Addgene (79057). Gateway full ORF clones encoding CSE and ETHE1 were obtained from the DKFZ Genomics & Proteomics Core Facility (Supplementary Table 1). All expression plasmids were constructed using the Gibson Assembly Cloning Kit (New England Biolabs). Primers for Gibson Assembly were designed using the NEBuilder Assembly tool. Expression plasmids used in this study were: pLPCX_APEX2-GFP, pLPCX_Su9-APEX2, pcDNA3.1_CSE, pcDNA3.1_ETHE1 and pTRC-APEX2.

### Antibodies

Primary antibodies: α-actin (A5441, Sigma-Aldrich), α-CSE (ab189916, Abcam), α-ETHE1 (GTX115707, GeneTex), α-GPX4 (ab125066, Abcam), α-MPST (PA5-51548, Thermo Fisher Scientific), α-SQR (ab71978, Abcam) and α-xCT (26864-1-AP, Proteintech). All primary antibodies were used at a dilution of 1:500, except for α-actin (1:1,000). HRP-conjugated antibodies: anti-mouse (115-035-146, Jackson ImmunoResearch) and anti-rabbit (111-035-144, Jackson ImmunoResearch). Secondary antibodies were used at a dilution of 1:10,000.

### Cell culture

HeLa (American Type Culture Collection (ATCC)), U2OS (ATCC), Phoenix-AMPHO (ATCC) and Pfa1 cells^22^ (a kind gift of Dr. Marcus Conrad, Helmholtz Munich) were maintained in DMEM (Life Technologies) supplemented with 10% (v/v) bovine calf serum (Life Technologies) and 50 U ml^−1^ of penicillin and streptomycin (Life Technologies). All cell lines were confirmed to be free of mycoplasma, viral infections and contaminations with other cell lines, based on multiplex polymerase chain reaction (PCR) and single nucleaotide polymorphism (SNP) profiling.

### Small interfering RNA–mediated depletion of enzymes

The small interfering RNA (siRNA) (ON-TARGETplus, SMARTpool, Horizon Discovery; Supplementary Table 1) was mixed with 3 µl of transfection reagent (DharmaFECT 1, Horizon Discovery) in a total volume of 500 µl of OptiMEM (Gibco) and incubated for 20 minutes at room temperature. The amount of siRNA used per transfection was: 20 pmol for CSE, 10 pmol for ETHE1, 10 pmol for MPST and 5 pmol for SQR. Non-targeting siRNA was used as a negative control. Meanwhile, cells were harvested with trypsin, diluted in antibiotics-free DMEM and counted. After adjusting the cell concentration to 37,000 cells per ml, 2 ml of cell suspension was added to the transfection mix into a well of a six-well plate. After 16 hours, the medium was replaced by fresh antibiotics-containing medium. Cells were used for analysis 72 hours after transfection.

### Overexpression of enzymes

In total, 250,000 cells per well were seeded into a six-well plate 1 day before transfection. For each transfection, 5 µl of Lipofectamine 2000 (11668019, Invitrogen) was added to 100 µl of OptiMEM (Gibco), vortexed briefly and incubated for 5 minutes at room temperature. In parallel, plasmid DNA (2.5 µg for CSE and 1 µg for ETHE1) was added to 100 µl of OptiMEM. Empty vectors were used as negative controls. The two solutions were combined and vortexed for 20 seconds. The mixture was incubated at room temperature for 25 minutes and then added to the cells. After 6 hours, the medium was replaced with fresh medium.

### Generation of HeLa cells stably expressing APEX2

For generation of stable cell lines, the pLPCX retroviral expression vector encoding APEX2-GFP or mito-APEX2 (Su9-APEX2) was transfected into the packaging cell line Phoenix-AMPHO. After 24 hours, viral supernatant was collected, filtered through a 0.45-μm cellulose acetate filter and used to infect freshly thawed HeLa cells. Transduced cells were selected with puromycin, expanded and frozen for later use. Cells expressing APEX2-GFP were enriched by fluorescence-activated cell sorting (FACS), and cells expressing mito-APEX2 were enriched by puromycin selection.

### Immunoblot analysis of protein expression

Cells were lysed with 0.1% Triton X-100 in TBS (10 mM Tris–HCl and 150 mM NaCl, pH 7.4) in the presence of protease inhibitors (complete, 04693132001, Roche). The lysate protein content was estimated using the BCA assay (23225, Thermo Fisher Scientific). Lysate samples were dissolved in SDS-PAGE sample buffer containing 10 mM DTT. Samples containing 20 µg of protein were run on an SDS-PAGE gel and transferred to polyvinyl difluoride (PVDF) membranes (Immobilon-F, Millipore) using a transfer tank (TE22, Hoefer). Membranes were probed with appropriate antibodies, HRP-conjugated secondary antibodies and chemiluminescent substrate (SuperSignal West Femto, Thermo Fisher Scientific).

### ATP-Glo bioluminometric cell viability assay

In total, 1,000 cells (Pfa1) or 10,000 cells (HeLa and U2OS) per well were seeded into white opaque 96-well plates (136101, Thermo Fisher Scientific). After 4 hours, cells were treated with 4-hydroxytamoxifen and other compounds as indicated in the figure legends. After 72 hours (Pfa1 xCT OE cells) or 20 hours (HeLa and U2OS cells), 50 µl of CellTiter-Glo 2.0 reagent (Promega, G9241) was added. Plates were shaken at 300 r.p.m. for 20 minutes on a rotary shaker (Titramax 101, Heidolph). Luminescence was recorded with a microplate reader (PHERAstar, BMG).

### PrestoBlue cell viability assay

In total, 1,000 cells per well were seeded into black 96-well plates with a transparent bottom (655090, Greiner). After 96 hours, 10 µl of the PrestoBlue reagent (A13261, Invitrogen) was added to each well, and plates were incubated at 37 °C for 20 minutes. Fluorescence (ex 545 nm/em 600 nm) was recorded with a microplate reader (CLARIOstar, BMG).

### Continuous cell viability measurements

In total, 10,000 cells (HeLa or U2OS) per well, in DMEM with 2% FCS and without phenol red, were seeded into black 96-well plates with a transparent bottom (655090, Greiner). The next day, cells were treated with RSL3 or CHP, as indicated in the figure legends. CellTox Green reagent (G8741, Promega) was added according to the manufacturer’s protocol. Green fluorescence (ex 480/em 520) was monitored at 37 °C (5% CO_2_) with a plate reader (CLARIOstar, BMG).

### Lipid aldehyde staining

In total, 30,000 (Pfa1 xCT OE) or 250,000 cells (HeLa) per well were seeded into six-well plates in DMEM with 10% FCS or in FluoroBrite with 2% FCS. After 44 hours (Pfa1 xCT OE) or 24 hours (HeLa), CHH (200 µM) and the corresponding treatment were added. Four hours later, cells were washed with PBS and detached with TrypLE (Gibco). Cells were spun down and resuspended in PBS. Gating on live cells (Supplementary Fig. 1), fluorescence was measured by flow cytometry (ex 405 nm/em 450 nm) l (FACSCanto II, BD). The protocol is based on ref. ^44^.

### BODIPY LPO assay

In total, 250,000 HeLa cells per well were seeded into six-well plates in DMEM (10% FCS) or FluoroBrite (2% FCS). After 24 hours, Bodipy C11 (Invitrogen) (10 µM) and the corresponding treatment were added. Four hours later, cells were washed once with PBS and detached with TrypLE (Gibco). Cells were spun down and resuspended in PBS. Gating on live cells (Supplementary Fig. 1), fluorescence was measured by flow cytometry (ex 488 nm/em 520 nm) (FACSCanto II, BD).

### S^0^ measurements

In total, 30.000 (Pfa1 xCT OE) or 250.000 cells (HeLa) per well were seeded into six-well plates. After 4 hours (Pfa1 xCT OE) or 24 hours (HeLa), cells were treated as indicated in the figure legends. Forty-seven hours later, the medium was aspirated, and SSP4 in PBS (10 µM final concentration) was added to the wells. After 30 minutes of incubation, cells were detached with TrypLE (Gibco), spun down and resuspended in PBS. Gating on live cells (Supplementary Fig. 1), fluorescent staining was measured by flow cytometry (ex 488 nm/em 520 nm) (FACSCanto II, BD).

### Measurement of sulfur-containing metabolites by ultra-performance liquid chromatography–mass spectroscopy

In total, 1 × 10^6^ cells (Pfa1 xCT OE) or 2 × 10^6^ cells (HeLa) were seeded in 10-cm dishes in DMEM with 10% FCS. Cells were washed twice with ice-cold 0.9% NaCl, followed by 270 µl of 50% H_2_O/MeOH with 5 mM MBB. Cells were scraped off the plate and transferred to Eppendorf tubes and then incubated for 20 minutes in the dark at room temperature. The cell suspension was spun down at 14,000*g* for 10 minutes, and 3 µl of supernatant was applied to an Accucore 150 Amide HILIC HPLC column (100 × 2.1 mm, 2.6-µm particle size) equipped with a guard cartridge (at 30 °C). Mobile phase ‘A’ was 5 mM ammonium acetate in 5% acetonitrile (CH_3_CN); mobile phase ‘B’ was 5 mM ammonium acetate in 95% CH_3_CN. The liquid chromatography (LC) gradient program was: 98% B for 1 minute, followed by a linear decrease to 40% B within 5 minutes, then maintaining 40% B for 13 minutes, then returning to 98% B in 1 minute and finally 5 minutes 98% B for column equilibration. The flow rate was 350 μl min^−1^. The eluent was directed to the electrospray ionization (ESI) source of the Q Exactive (QE) MS from 0.5 minutes to 19 minutes after sample injection. Each sample was run with the full MS method for general metabolites and with the parallel reaction monitoring (PRM) method for bromobimane alkylated metabolites. Full MS method: scan type: full MS in positive/negative mode with ddMS^2^; runtime: 0.5‒19 minutes; resolution: 70,000; AGC target: 1 × 10^6^; maximum injection time: 50 ms; scan range: 69‒1,000 *m*/*z*; sheath gas flow rate: 30; auxiliary gas flow rate: 10; sweep gas flow rate: 0; spray voltage: 3.6 kV (positive)/2.5 kV (negative); capillary temperature: 320 °C; S-lens RF level: 55.0; auxiliary gas heater temperature: 120 °C. ddMS^2^ settings: resolution: 17,500; AGC target: 1 × 10^5^; maximum injection time: 50 ms; loop count: 1; CE: 20, 50 and 80; apex trigger: 0.1‒10 seconds; minimum AGC target: 2.00 × 10^3^; dynamic exclusion: 20 seconds. PRM method: scan type: PRM positive mode; runtime: 0.5‒10 minutes. ddMS^2^ settings: resolution: 17,500; AGC target: 2 × 10^5^; maximum injection time: 200 ms; loop count: 1; CE: 20, 50 and 80; isolation window: 1.2 *m*/*z*. For the transition list, see Supplementary Table 2. For normalization among the samples, at least 20 core metabolites (amino acids and nucleotides) were quantified from each sample in negative and positive mode with El-MAVEN (0.12.0)^45^. PRM data were processed with Skyline (21.2.0.425)^46^ and normalized to metabolite data.

### Real-time monitoring of intracellular radical load

In total, 25,000 APEX2-expressing HeLa cells, suspended in FluoroBrite (Gibco, A1896701) with 2% FCS, were seeded per well into opaque white 96-well plates (Thermo Fisher Scientific, 136101). Stock solutions were prepared by dissolving luminol (2.5 mM) in 50 mM borate buffer (H_3_BO_3_, pH 9) and the peroxidase substrate HPI (2.5 mM) in PBS with 30% DMSO. After 18 hours, luminol and HPI were added to the wells, both to 250 µM final concentration. Luminescence was recorded with a microplate reader (PHERAstar, BMG). Twenty minutes after the start of the measurement, 50 µM of H_2_O_2_ was added. After the measurement, cells were lysed with 0.1% Triton X-100 and stained with SYBR Green (S9430, Sigma-Aldrich) in the well. SYBR Green fluorescence (ex 480 nm/em 520 nm) was recorded with a microplate reader (PHERAstar, BMG). Luminescence was normalized to SYBR fluorescence. For quantitative comparisons of intracellular radical load, the area under the curve (AUC) was calculated with GraphPad Prism 8.

### Expression and purification of recombinant APEX2

APEX2 was expressed from a plasmid (72558 pTRC-APEX2, Addgene) in *Escherichia coli* BL21(DE3) (EC0114, Thermo Fisher Scientific), as described previously^23^. In brief, 500 ml of Luria broth (LB) with 10 μg ml^−1^ of ampicillin was inoculated with a single colony. The culture was grown at 37 °C to an OD_600_ of 1. Then, protein expression was induced with IPTG (420 μM). 5-aminolevulinic acid hydrochloride (1 mM) was added to promote heme biosynthesis. The culture was continued overnight at room temperature and then centrifuged for 10 minutes at 4,000 relative centrifugal force (RCF) and 4 °C. The dried bacterial pellet was solubilized in 20 ml of B-PER (78243, Thermo Fisher Scientific), supplemented with protease inhibitors (1 µg ml^−1^ of leupeptin, 1 µg ml^−1^ of AEBSF HCl and 1 µl of benzonase) and 5 mM imidazole and then transferred to a 50-ml centrifuge tube. The lysate was carefully mixed for 10 minutes at 4 °C and then centrifuged at 16,000 RCF for 30 minutes at 4 °C. The supernatant was collected. Ni-NTA agarose beads (30210, Qiagen) were washed three times with NH_4_HCO_3_ buffer (50 mM NH_4_HCO_3_, pH 7.4). The bacterial lysis supernatant was added to 1 ml of Ni-NTA agarose beads and incubated for 1 hour at 4 °C under gentle rotation. The Ni-NTA bead suspension was transferred to 5-ml disposable chromatography columns (29922, Thermo Fisher Scientific). Beads were washed three times with 5 ml of wash buffer (50 mM NaH_2_PO_4_, 300 mM NaCl, 20 mM imidazole, 1 µg ml^−1^ of leupeptin, 1 µg ml^−1^ of AEBSF HCl and 1 µl benzonase, pH 8.0). The protein was eluted in three steps, first with 5 ml of elution buffer containing 50 mM imidazole (50 mM NaH_2_PO_4_, 300 mM NaCl and 50 mM imidazole, pH 8.0) and then with 5 ml of elution buffer containing 100 mM imidazole and finally with 5 ml of elution buffer containing 500 mM of imidazole. Eluates containing pure protein (as determined by SDS-PAGE) were combined. The combined protein sample was placed in a 10-kDa cutoff dialysis cassette (66382, Thermo Fisher Scientific) pre-equilibrated with PBS buffer for 5 minutes. The cassette was then incubated in 2 L of PBS buffer under constant stirring. Dialysis was performed overnight at 4 °C. Finally, 1-ml aliquots of dialyzed protein were stored at −80 °C. The final protein concentration was estimated using the BCA assay.

### Electron spin resonance spectroscopy

For the analysis of in vitro samples, reactants, including the spin trap, were mixed in PBS containing diethylenetriaminepentaacetic acid (DTPA, 50 µM) to a final volume of 50 µl, using low protein binding tubes (90410, Thermo Fisher Scientific). For the analysis of intact cells, 5 × 10^6^ cells were resuspended in PBS containing 50 µM DTPA, using low protein binding tubes (90410, Thermo Fisher Scientific). The spin trap and other reagents (peroxidase substrate, S^0^ species and H_2_O_2_) were added as described in the figure legends to a final volume of 50 µl. Samples were transferred to capillaries (ring caps, NOXygen), sealed with Critoseal (Noxygen) and immediately inserted into the cavity (ST9010) of the electron paramagnetic resonance (EPR) spectrometer (ESP300e, Bruker). Spectra were recorded and pre-processed with proprietary software (Lila-X and Medeia, provided by Gerhard Bracic). Spectrometer settings: microwave attenuation 10 dB (=20 mW microwave power); modulation amplitude 0.1 mT; receiver gain 60 dB; modulation frequency 100 kHz; conversion time 40 ms; time constant 20 ms; center field 339.5 mT; sweep width 14.0 mT. The microwave frequency was measured with an HP 5010 frequency counter. When necessary, spectra were accumulated to improve the signal-to-noise ratio. Control experiments were run with different combinations of reactants to identify unwanted side reactions. Spectra were simulated with the EasySpin package for MATLAB^47^.

### Cyclic voltammetry

Ferrocene (0.5 mM) was mixed with GSH and GSSSG in N_2_-saturated Britton–Robinson buffer (0.04 M boric acid, 0.04 M phosphoric acid and 0.04 M acetic acid) at pH 9. Measurements were conducted with an Autolab PGSTAT12 potentiostat–galvanostat electrochemical system (Eco Chemie) using a conventional three-electrode setup. An Ag/AgCl (3 M KCl) electrode was used as a reference electrode, and a platinum wire served as the counter electrode. The working electrode was a glassy carbon electrode (Metrohm, 6.1204.300) with a diameter of 3 mm. It was cleaned before each experiment by polishing it with aluminum oxide powder (grain size 0.3 µm) (Alfa Aesar, 14558) for 30 seconds and then rinsing it with ethanol and deionized water. The potential step was 1 mV, and the scan rate was 10 mV s^−1^ or 20 mV s^−1^. Measurements were carried out in de-aerated (N_2_-saturated) solutions at room temperature under quiescent conditions. Data were analyzed with General Purpose Electrochemical System (GPES) version 4.9 (Eco Chemie) software.

### Amplex Red autoxidation assay

Amplex Red autoxidation (resorufin formation) was measured as described previously^24^. Reactants (as indicated in the figure legends) were mixed in PBS. Resorufin formation was monitored in 96-well plates by measuring absorbance at 562 nm (FLUOstar, BMG) or fluorescence (ex 571 nm/em 585 nm) (CLARIOstar, BMG) at room temperature.

### Cyt c reduction assay

Ferric cyt c (24 µM) was mixed with GSH (1 mM) and variable amounts of polysulfides (as indicated in the figure legend) in PBS. Absorbance at 550 nm was recorded with a microplate reader (FLUOstar, BMG) at room temperature.

### TEMPOL reduction assay

TEMPOL (18 mM) was mixed with GSH (20 mM) and variable amounts of CSSSC (as indicated in the figure legend) in PBS containing DTPA (50 µM). Absorbance at 430 nm was recorded with a microplate reader (FLUOstar, BMG) at room temperature.

### Analysis of GSSSSG generation

Ferric cyt c (400 µM) or ABTS (400 µM) was mixed with GSSSG or GS^34^SSG (400 µM), NADPH (400 µM) and GR (1 U ml^−1^) in a total volume of 100 µl in PBS. After 5 minutes, the reaction was stopped by the addition of 100 µl of MBB in MeOH (10 mM). The samples were incubated for 5 minutes in the dark, and then 200 µl of CHCl_3_ was added to remove GR by precipitation. After centrifugation at 300 RCF, the upper phase was removed for further analysis. The sample (10 µl) was injected into an Agilent 1260 Infinity LC system attached to an Agilent 6120 Single Quadrupole MS with ESI source and evaporative light scattering detector (ELSD). Separation was performed on a Kinetex 2.6 μm C18 100 Å LC column (50 × 2.1 mm) at 40 °C using a flow rate of 0.6 ml min^−1^. Solvent ‘A’ was 0.01% HCOOH in water; solvent ‘B’ was 0.01% HCOOH in MeCN. The method was: 100% A for 2 minutes, then from 100% to 10% A in 10 minutes and then 1% A for another 10–12 minutes. Data were processed with MestReNova (14.2.1) software.

### Electrode measurement of H_2_S

Reactions were performed in 1 ml of PBS containing 1 mM of GSH, 50 µM of ferric cyt c and 10 µM of GSSSG. H_2_S release was recorded with a hydrogen sulfide electrode (TBR 4100, WPI) by submerging the sensor tip into the well of a 12-well plate. The plate was agitated on a rotary shaker (Titramax 101) with 150 r.p.m. to prevent the buildup of a diffusion layer around the electrode. Data were analyzed with LabScribe software.

### Synthesis of CSSSC

CSSSC was synthesized as described previously^48^. In brief, 1 g of cystine (CSSC) was dissolved in 1 M H_2_SO_4_ and reacted with 2 equivalents of peracetic acid on ice for 2 hours. The product cystine-S-oxide was precipitated by neutralizing the solution with pyridine and thoroughly washed with EtOH and THF. Cystine-S-oxide was redissolved in 1 M H_2_SO_4_ and reacted with 2 equivalents of NaSH for 2 hours at room temperature. The final product CSSSC was precipitated by neutralizing the solution with pyridine, thoroughly washed with EtOH and THF and then lyophilized, with a yield of approximately 25%. High-performance liquid chromatography–mass spectroscopy (HPLC–MS) analysis indicated >98% purity (Supplementary Note 1).

### QM calculations

Geometries were optimized, and Gibbs free energies were calculated with Gaussian 09 (ref. ^49^), using 6-31 + G(d) as the basis set. The Polarizable Continuum Model with water was used as the solvent model.

### Reporting summary

Further information on research design is available in the Nature Research Reporting Summary linked to this article.

## Online content

Any methods, additional references, Nature Research reporting summaries, source data, extended data, supplementary information, acknowledgements, peer review information; details of author contributions and competing interests; and statements of data and code availability are available at 10.1038/s41589-022-01145-w.

## Supplementary information

Supplementary Information Supplementary Tables 1 and 2, Supplementary Figs. 1 and 2 and Supplementary Note 1

Reporting Summary

## Data Availability

All data generated and analyzed in this study are included in this article and its Supplementary Information files. Source data are provided with this paper.

## Source data

Fig. 1 Source data
Fig. 2 Source data
Fig. 3 Source data
Fig. 4 Source data
Fig. 5 Source data
Fig. 6 Source data
Extended Data Fig. 1 Source data
Extended Data Fig. 1 Source data
Extended Data Fig. 2 Source data
Extended Data Fig. 2 Source data
Extended Data Fig. 3 Source data
Extended Data Fig. 3 Source data
Extended Data Fig. 4 Source data
Extended Data Fig. 5 Source data
Extended Data Fig. 6 Source data
Extended Data Fig. 7 Source data
Extended Data Fig. 8 Source data
Extended Data Fig. 9 Source data
