# Supplementary material for: Hydropersulfides inhibit lipid peroxidation and ferroptosis by scavenging radicals
Source: Nat Chem Biol. 2022 Sep 15;19(1):28–37. doi: 10.1038/s41589-022-01145-w (PMC7613997; doi:10.1038/s41589-022-01145-w)

nature portfolio

https://doi.org/10.1038/s41589-022-01145-w

Supplementary information

Hydropersulfides inhibit lipid peroxidation and ferroptosis by scavenging radicals

In the format provided by the authors and unedited

SUPPLEMENTARY INFORMATION

Persulfides inhibit lipid peroxidation and ferroptosis by scavenging free radicals

Uladzimir Barayeu[1,2], Danny Schilling[1,2,+], Mohammad Eid[1,2,+], Thamara Nishida Xavier da Silva[3], Lisa Schlicker[4,5], Nikolina Mitreska[6], Christopher Zapp[7], Frauke Gräter[7], Aubry K. Miller[8], Reinhard Kappl[6], Almut Schulze[4], José Pedro Friedmann Angeli[3], Tobias P. Dick[1,2,*]

[1]Division of Redox Regulation, German Cancer Research Center (DKFZ), Heidelberg, Germany

[2]Faculty of Biosciences, Heidelberg University, Heidelberg, Germany

[3]Rudolf-Virchow-Zentrum - Center for Integrative and Translational Bioimaging, University of Würzburg, Würzburg, Germany

[4]Division of Tumor Metabolism and Microenvironment, German Cancer Research Center (DKFZ), Heidelberg, Germany

[5]Proteomics Core Facility, German Cancer Research Center (DKFZ), Heidelberg, Germany

[6]Department of Biophysics, Faculty of Medicine, Center for Integrative Physiology and Molecular Medicine (CIPMM), Saarland University, Homburg, Germany

[7]Molecular Biomechanics, Heidelberg Institute for Theoretical Studies (HITS), Heidelberg, Germany

[8]Research Group Cancer Drug Development, German Cancer Research Center (DKFZ), Heidelberg, Germany

[+]Equal contribution
*Corresponding author

CONTENTS

- **Supplementary Table 1: Plasmids and siRNAs used in this study**
- **Supplementary Table 2: MS/MS transition list for per/polysulfides**
- **Supplementary Figure 1: FACS gating strategy**
- **Supplementary Figure 2: MS-based detection of isotopically labeled GSSSSG**
- **Supplementary Note 1: LC/MS-based characterization of synthesized CSSSC**

Supplementary Table 1. Plasmids and siRNAs used in this study

Plasmids:	
APEX2	72558, pTRC-APEX2, Addgene
CSE	114777419, GPCF, DKFZ
ETHE	194472089, GPCF, DKFZ
pMD2.G	12259, Addgene

siRNAs:	
CSE	003481, ON-TARGETplus, SMARTpool, Horizon Discovery
ETHE1	012508, ON-TARGETplus, SMARTpool, Horizon Discovery
SQR	008271, ON-TARGETplus, SMARTpool, Horizon Discovery
MST	010119, ON-TARGETplus, SMARTpool, Horizon Discovery

Supplementary Table 2. MS/MS transition list for per/polysulfides

**Metabolite**	**MBB derivative**	**Parent ion ($M+H^+$)**	**Fragment ion ($M+H^+$)**
CSSSC	-	273,0032	122,0274
GSSSG	-	645,1313	387,0471
GSSSSG	-	677,1034	372,0139
H_2S	Bim-S-Bim	415,1435	193,0971
H_2S_2	Bim-SS-Bim	447,1155	192,0893
GSH	Bim-SG	498,1653	225,0693
GSSH	Bim-SSG	530,1374	192,0893
GSSSH	Bim-SSSG	562,1095	192,0891
Cys	Bim-Cys	312,1013	192,089
CysSH	Bim-SCys	344,0733	192,089

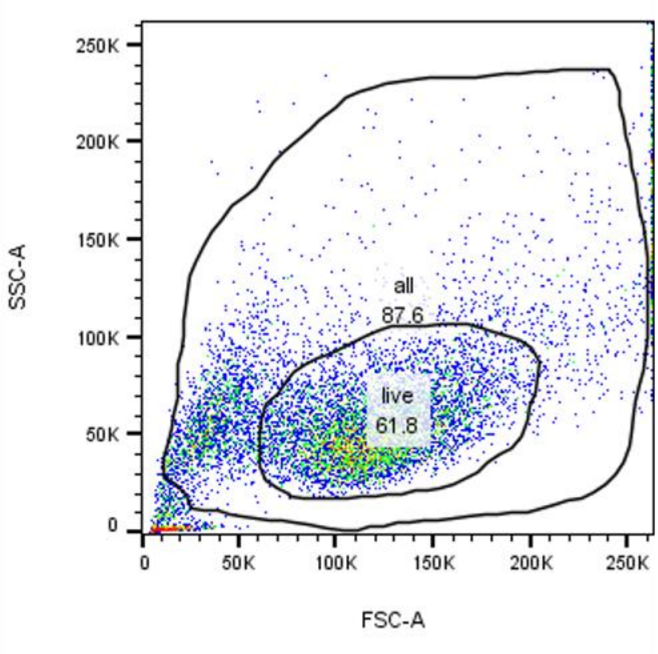

Supplementary Figure 1. FACS gating strategy
Gating of the live cell population is indicated.

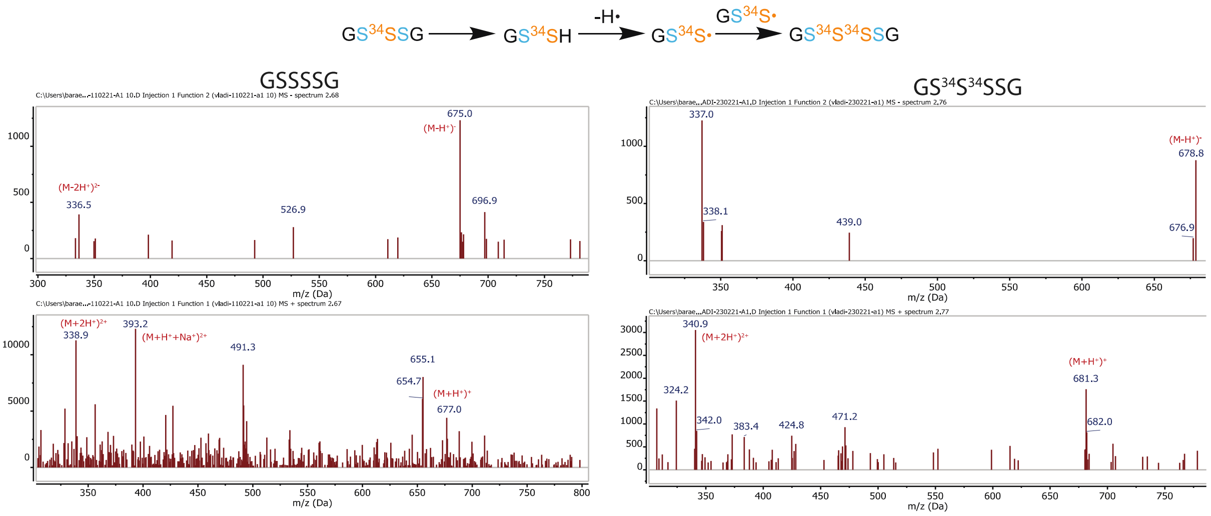

Supplementary Figure 2. MS-based detection of isotopically labeled GSSSSG.

The reaction scheme on top explains the generation of doubly-labeled GS^{34}S^{34}SSG from singly-labeled GS^{34}SSG. GS^{34}SSG is reduced to GS^{34}SH, which then reacts with a radical to form GS^{34}S・. The perthiyl radical self-recombines to generate GS^{34}S^{34}SSG. Mass spectra of unlabeled GSSSSG (left panels) and doubly-labeled GS^{34}S^{34}SSG (right panels) formed in the reaction of ferric cytochrome c with GSSH and GS^{34}SH, respectively. Negative (upper panels) and positive ion mode (lower panels).

Supplementary Note 1. LC/MS-based characterization of synthesized CSSSC

Cysteine trisulfide (CSSSC)

HPLC chromatogram

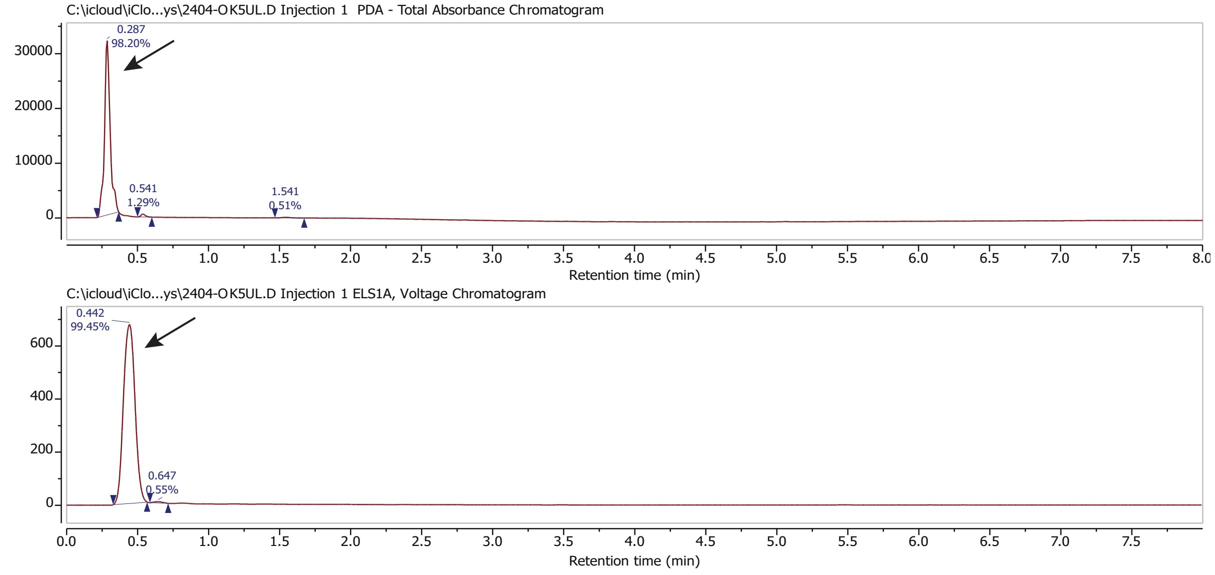

MS spectrum of the indicated peak

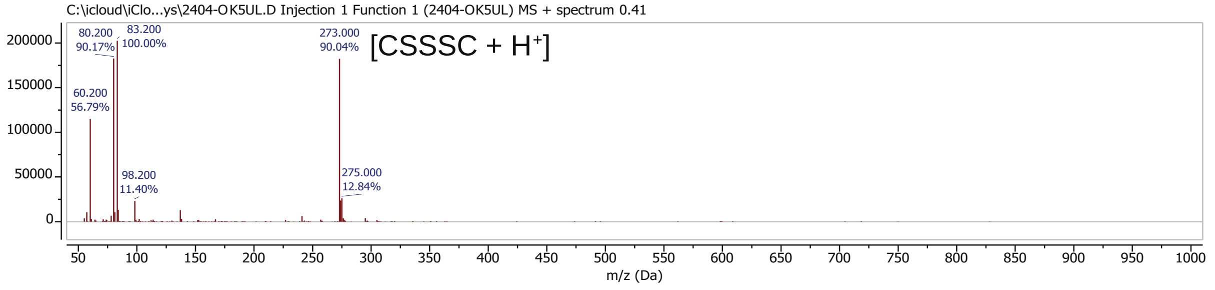

MS^2 spectrum

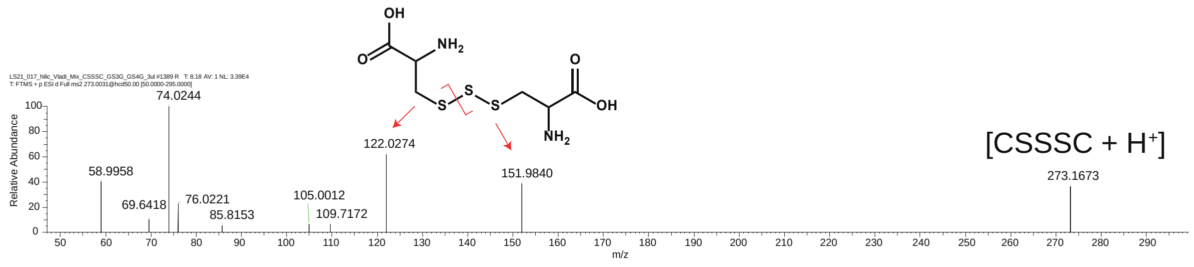

Supplement: Supplementary file 1 — Supplementary Tables 1 and 2, Supplementary Figs. 1 and 2 and Supplementary Note 1 [file 41589_2022_1145_MOESM1_ESM.pdf]
