# Supplementary material for: Hydropersulfides inhibit lipid peroxidation and ferroptosis by scavenging radicals
Source: Nat Chem Biol. 2022 Sep 15;19(1):28–37. doi: 10.1038/s41589-022-01145-w (PMC7613997; doi:10.1038/s41589-022-01145-w)
Supplement: Supplementary file 2 — Reporting Summary [file 41589_2022_1145_MOESM2_ESM.pdf]

Corresponding author(s): Tobias P. Dick

Last updated by author(s): Aug 11, 2022

Reporting Summary

Nature Research wishes to improve the reproducibility of the work that we publish. This form provides structure for consistency and transparency in reporting. For further information on Nature Research policies, see our Editorial Policies and the Editorial Policy Checklist.

Statistics

For all statistical analyses, confirm that the following items are present in the figure legend, table legend, main text, or Methods section.

n/a	Confirmed	
☐	☒	The exact sample size (*n*) for each experimental group/condition, given as a discrete number and unit of measurement
☐	☒	A statement on whether measurements were taken from distinct samples or whether the same sample was measured repeatedly
☐	☒	The statistical test(s) used AND whether they are one- or two-sided<br>*Only common tests should be described solely by name; describe more complex techniques in the Methods section.*
☒	☐	A description of all covariates tested
☒	☐	A description of any assumptions or corrections, such as tests of normality and adjustment for multiple comparisons
☐	☒	A full description of the statistical parameters including central tendency (e.g. means) or other basic estimates (e.g. regression coefficient) AND variation (e.g. standard deviation) or associated estimates of uncertainty (e.g. confidence intervals)
☐	☒	For null hypothesis testing, the test statistic (e.g. F, t, r) with confidence intervals, effect sizes, degrees of freedom and P value noted<br>*Give P values as exact values whenever suitable.*
☒	☐	For Bayesian analysis, information on the choice of priors and Markov chain Monte Carlo settings
☒	☐	For hierarchical and complex designs, identification of the appropriate level for tests and full reporting of outcomes
☒	☐	Estimates of effect sizes (e.g. Cohen's *d*, Pearson's *r*), indicating how they were calculated

Our web collection on statistics for biologists contains articles on many of the points above.

Software and code

Policy information about availability of computer code

Data collection	PHERAstar and CLARIOstar (v. 4.00 and v. 5.20, BMG), FACSDiva (v. 9, BD), Lila-X and Medeia (provided by Gerhard Bracic), Xcalibur (v. 4.3, Thermo Fisher), Gaussian09.
Data analysis	Mars (BMG), FlowJo (v. 10.8, BD Life Sciences), El-MAVEN (v. 0.12.0), Skyline (v. 21.2.0.425), GraphPad Prism (v. 8), Easyspin package for MatLab R2021a, MestReNova (v. 14.2.1, Mestrelab), General Purpose Electrochemical System (v. 4.9, Eco Chemie B. V.), Labscribe (v. 4, WPI).

For manuscripts utilizing custom algorithms or software that are central to the research but not yet described in published literature, software must be made available to editors and reviewers. We strongly encourage code deposition in a community repository (e.g. GitHub). See the Nature Research guidelines for submitting code & software for further information.

Data

Policy information about availability of data

All manuscripts must include a data availability statement. This statement should provide the following information, where applicable:
- Accession codes, unique identifiers, or web links for publicly available datasets
- A list of figures that have associated raw data
- A description of any restrictions on data availability

All data generated and analyzed in this study are included in this article and its supplementary information files.

Field-specific reporting

Please select the one below that is the best fit for your research. If you are not sure, read the appropriate sections before making your selection.

☒ Life sciences ☐ Behavioural & social sciences ☐ Ecological, evolutionary & environmental sciences

For a reference copy of the document with all sections, see nature.com/documents/nr-reporting-summary-flat.pdf

Life sciences study design

All studies must disclose on these points even when the disclosure is negative.

Sample size	Generally, experiments involving cells were repeated independently on different days using cells of different passage number at least 3 times (biological replicates). For each biological replicate the mean derived from technical replicates (n=3-6) is shown as an individual data point. Some experiments involving cells (LC/MS, viability and luminescence measurements) were repeated independently at least 3 times on the same day using separate cell cultures of the same passage number. For ESR spectra, FACS histograms and luminescence curves representative results are shown. In vitro chemistry experiments were repeated at least 2 times. For ESR spectra, cyclic voltammetry data and APEX spectroscopy data representative experiments are shown.
Data exclusions	No data were excluded from analysis.
Replication	All attempts at replication were successful.
Randomization	No experimental groups were used in this study. Randomization therefore was not relevant.
Blinding	Group allocation and blinding was not relevant in this study.

Reporting for specific materials, systems and methods

We require information from authors about some types of materials, experimental systems and methods used in many studies. Here, indicate whether each material, system or method listed is relevant to your study. If you are not sure if a list item applies to your research, read the appropriate section before selecting a response.

Materials & experimental systems

n/a	Involved in the study	
☐	☒	Antibodies
☐	☒	Eukaryotic cell lines
☒	☐	Palaeontology and archaeology
☒	☐	Animals and other organisms
☒	☐	Human research participants
☒	☐	Clinical data
☒	☐	Dual use research of concern

Methods

n/a	Involved in the study	
☒	☐	ChIP-seq
☐	☒	Flow cytometry
☒	☐	MRI-based neuroimaging

Antibodies

Antibodies used	The following antibodies were used: α-actin (A5441, Sigma), α-CSE (ab189916, Abcam), α-ETHE1 (GTX115707, Genetex), α-GPX4 (ab125066, Abcam), α-MPST (PA5-51548, ThermoFisher), α-SQR (ab71978, Abcam) and α-xCT (26864-1-AP, Proteintech). All primary antibodies were used at 1:500 dilution, except for α-actin (1:1000). HRP-conjugated antibodies: anti-mouse (115-035-146, Jackson ImmunoResearch), anti-rabbit (111-035-144, Jackson ImmunoResearch). HRP-conjugated antibodies were used at 1:10.000 dilution.
Validation	Validation statements for each primary antibody are provided on the manufacturer's website. Additionally, primary antibodies were validated by the knockdown and over-expression experiments shown in this study.

Eukaryotic cell lines

Policy information about cell lines

Cell line source(s)	HeLa, U2OS and Phoenix Ampho cells were purchased from ATCC. Pfa1 cells were a kind gift from Dr. Marcus Conrad (Helmholtz Center Munich).
Authentication	Cell lines were authenticated via SNP-based Multiplex Cell Line Authentication (www.multiplexion.de).
Mycoplasma contamination	Cell cultures were repeatedly tested negative for Mycoplasma contamination by PCR.

Commonly misidentified lines (See ICLAC register)	No commonly misidentified cell lines were used.

Flow Cytometry

Plots

Confirm that:

☒ The axis labels state the marker and fluorochrome used (e.g. CD4-FITC).

☒ The axis scales are clearly visible. Include numbers along axes only for bottom left plot of group (a 'group' is an analysis of identical markers).

☒ All plots are contour plots with outliers or pseudocolor plots.

☒ A numerical value for number of cells or percentage (with statistics) is provided.

Methodology

Sample preparation	Cells were washed with PBS and then detached with TrypLE (Gibco). Cells were collected by centrifugation and resuspended in PBS.
Instrument	FACS Canto II (BD)
Software	FlowJo™ v10.8 Software (BD Life Sciences)
Cell population abundance	At least 10.000 cells were measured. The live cell population was at least 4000 cells.
Gating strategy	Forward versus side scatter (FSC vs. SSC) was used to gate on live single cells.

☒ Tick this box to confirm that a figure exemplifying the gating strategy is provided in the Supplementary Information.
