# Supplementary figures and images for: Hydropersulfides inhibit lipid peroxidation and ferroptosis by scavenging radicals
Source: Nat Chem Biol. 2022 Sep 15;19(1):28–37. doi: 10.1038/s41589-022-01145-w (PMC7613997; doi:10.1038/s41589-022-01145-w)

GPX4 and actin WB

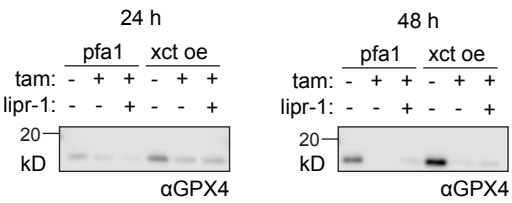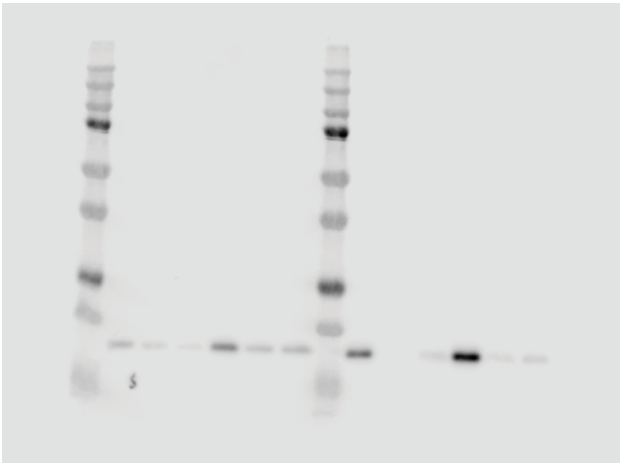

αGPX4

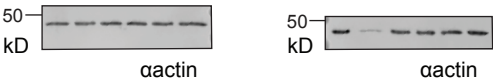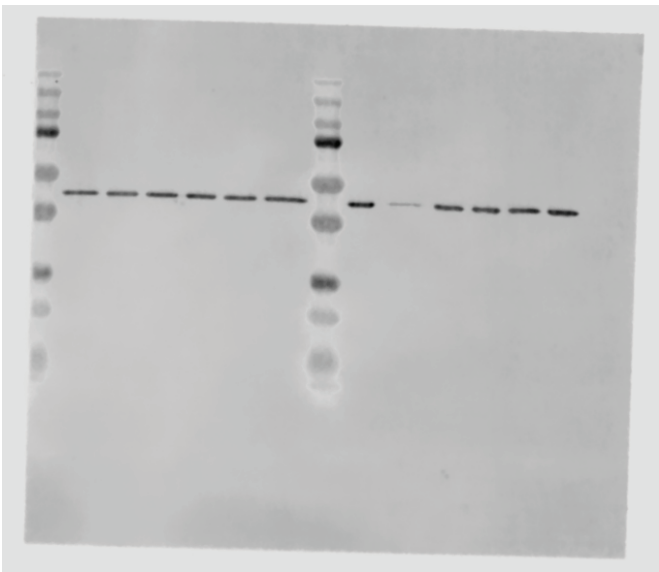

αactin

Supplement: Extended Data Fig. 1 — Source data [file 41589_2022_1145_MOESM10_ESM.zip › Ex.Fig1/WB_1_Ex.Fig.1E.pdf]

# xCT WB

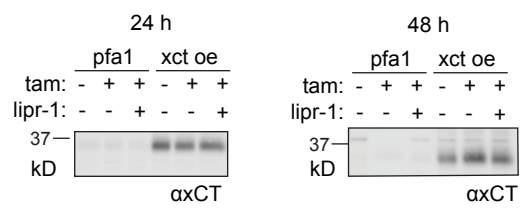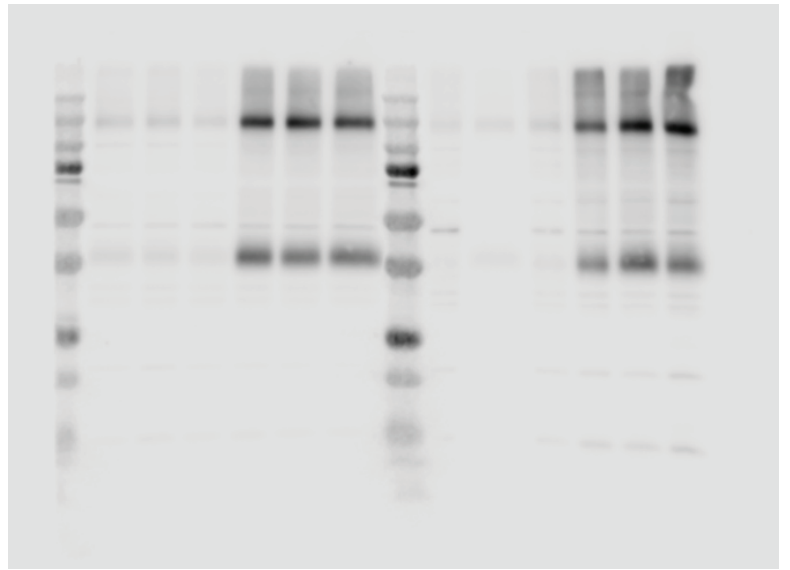

αxCT

Supplement: Extended Data Fig. 1 — Source data [file 41589_2022_1145_MOESM10_ESM.zip › Ex.Fig1/WB_2_Ex.Fig.1E.pdf]

# CSE WB

|             | siRNA |   |
|-------------|-------|---|
| non-target: | +     | - |
| CSE:        | -     | + |

50  
kD

αCSE

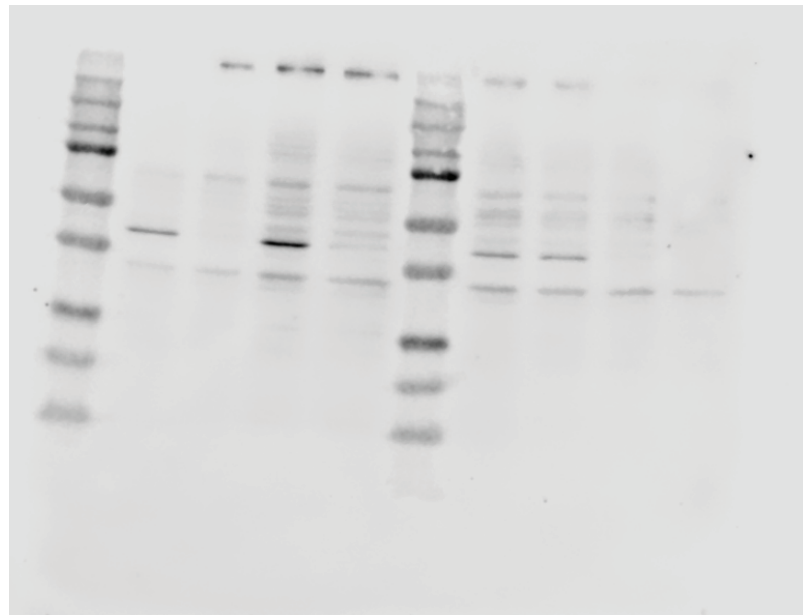

αCSE

50  
kD

αactin

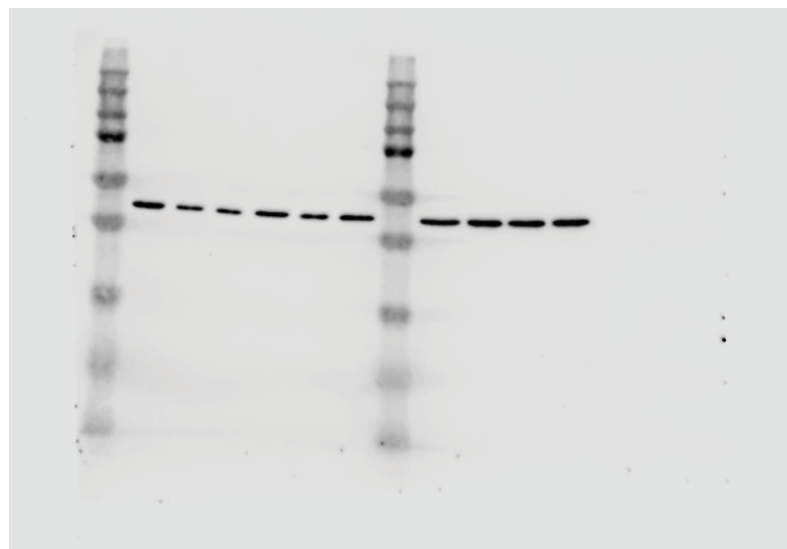

αactin

Supplement: Extended Data Fig. 2 — Source data [file 41589_2022_1145_MOESM12_ESM.zip › Ex.Fig2/WB_3_Ex.Fig.2B.pdf]

# CSE WB

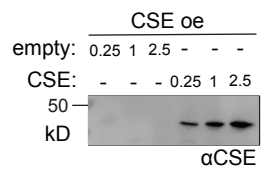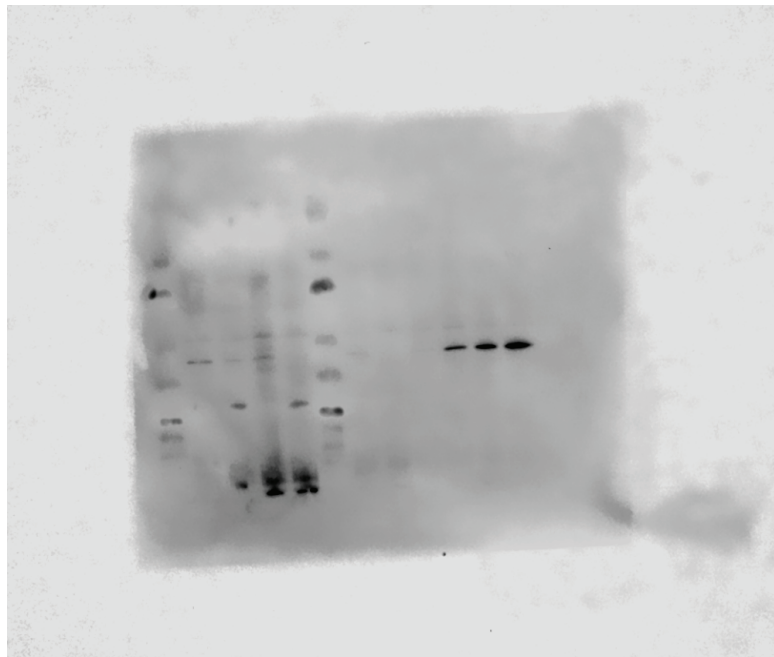

$\alpha$ CSE

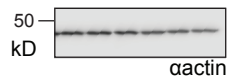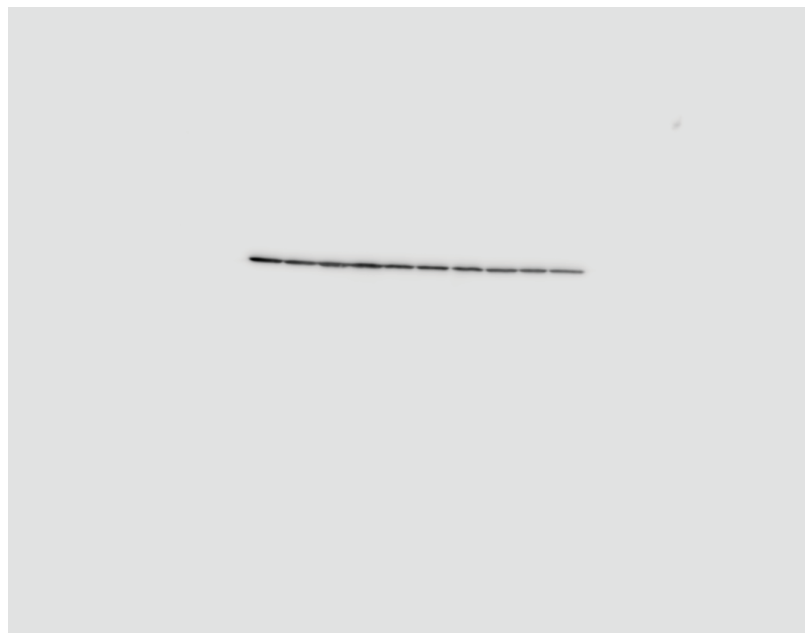

$\alpha$ actin

Supplement: Extended Data Fig. 2 — Source data [file 41589_2022_1145_MOESM12_ESM.zip › Ex.Fig2/WB_4_Ex.Fig.2D.pdf]

ETHE1 WB

|             |       |   |
|-------------|-------|---|
|             | siRNA |   |
| non-target: | +     | - |
| ETHE1:      | -     | + |

25  
kD

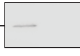

αETHE1

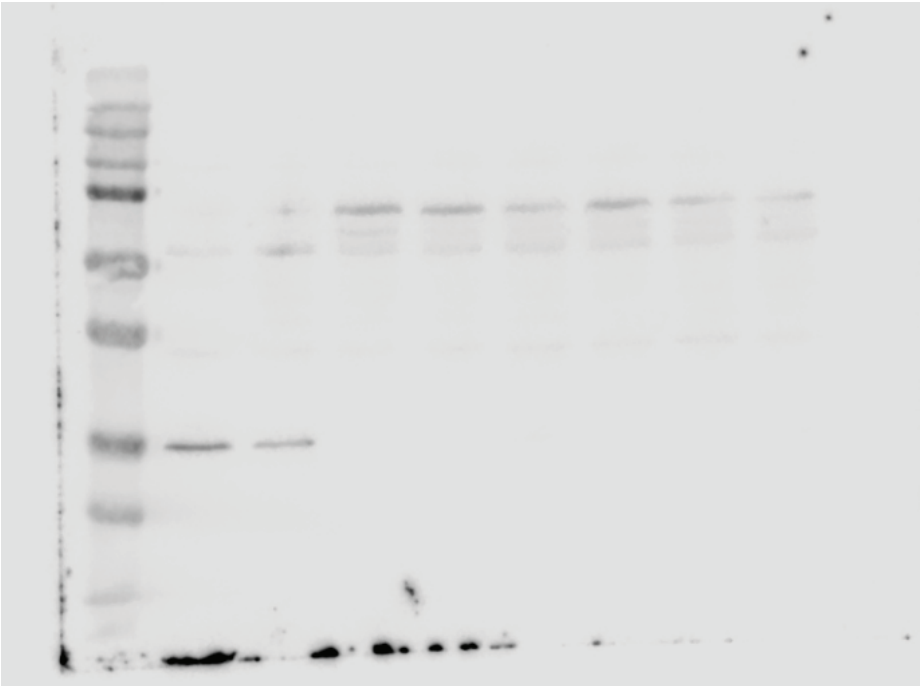

αETHE1

50  
kD

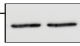

αactin

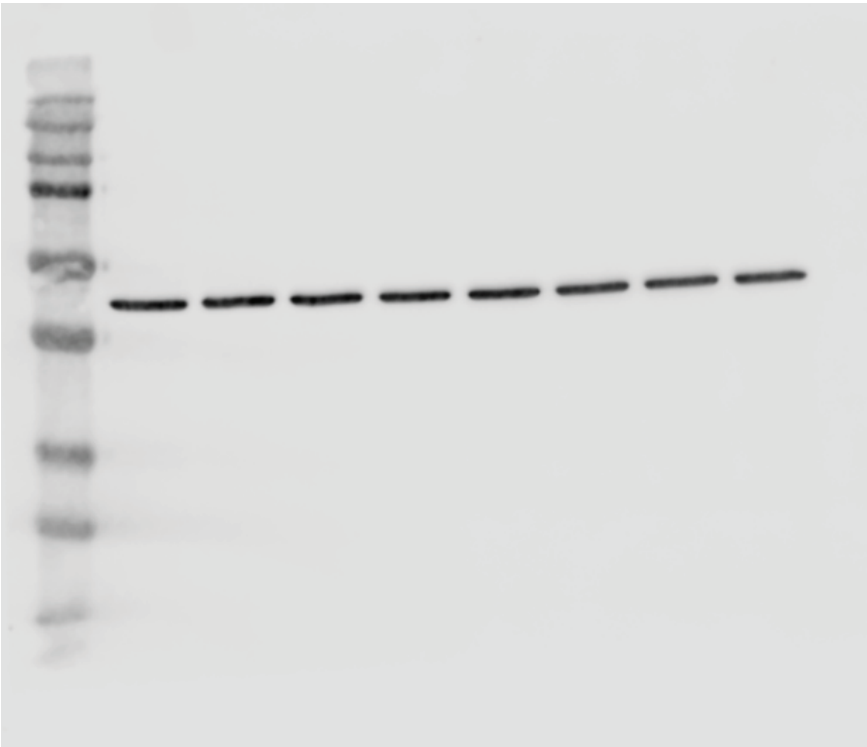

αactin

Supplement: Extended Data Fig. 3 — Source data [file 41589_2022_1145_MOESM14_ESM.zip › Ex.Fig3/WB_5_Ex.Fig.3C.pdf]

# ETHE1 WB

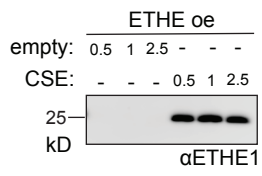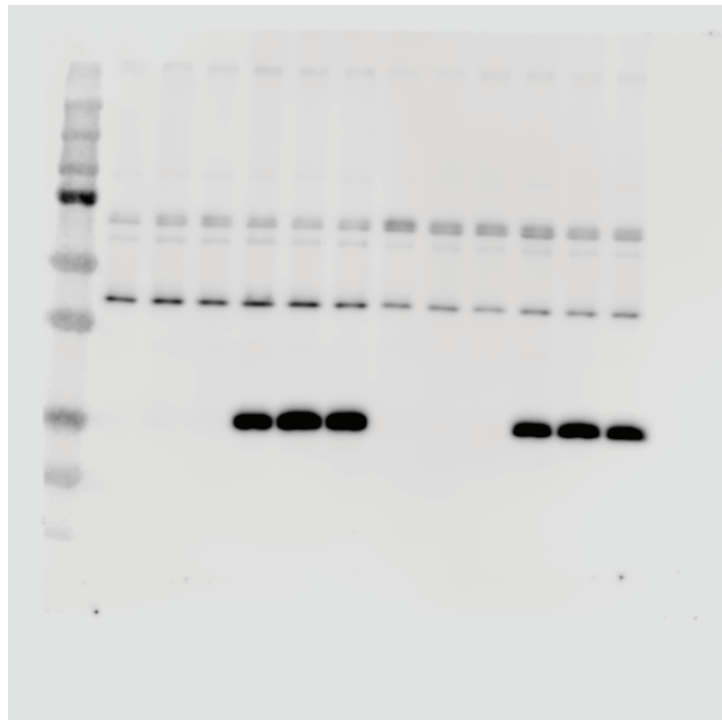

$\alpha$ ETHE1

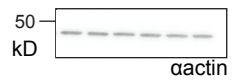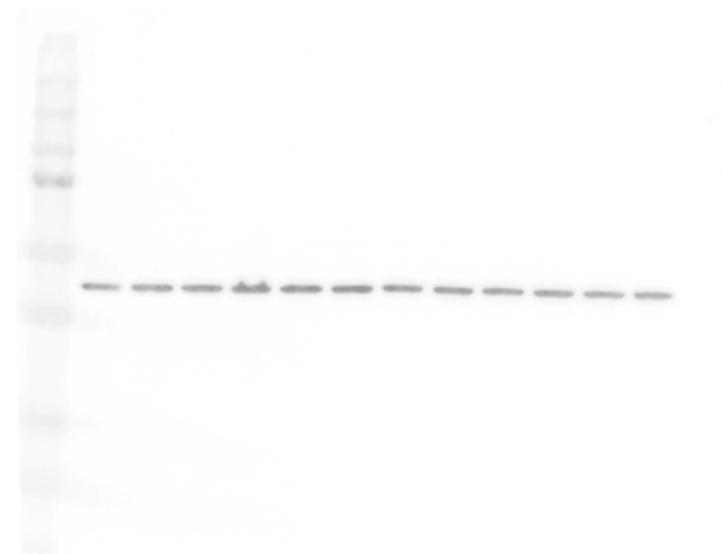

$\alpha$ actin

Supplement: Extended Data Fig. 3 — Source data [file 41589_2022_1145_MOESM14_ESM.zip › Ex.Fig3/WB_6_Ex.Fig.3E.pdf]

# SQR WB

|             |                                                                                   | SQR kd |     |   |    |   |
|-------------|-----------------------------------------------------------------------------------|--------|-----|---|----|---|
| non-target: | +                                                                                 | -      | -   | - | -  | - |
| SQR:        | -                                                                                 | 1.25   | 2.5 | 5 | 10 |   |
| 50          | 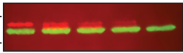 |        |     |   |    |   |
| 37          |                                                                                   |        |     |   |    |   |
| kD          | $\alpha$ SQR (red)<br>$\alpha$ actin (green)                                      |        |     |   |    |   |

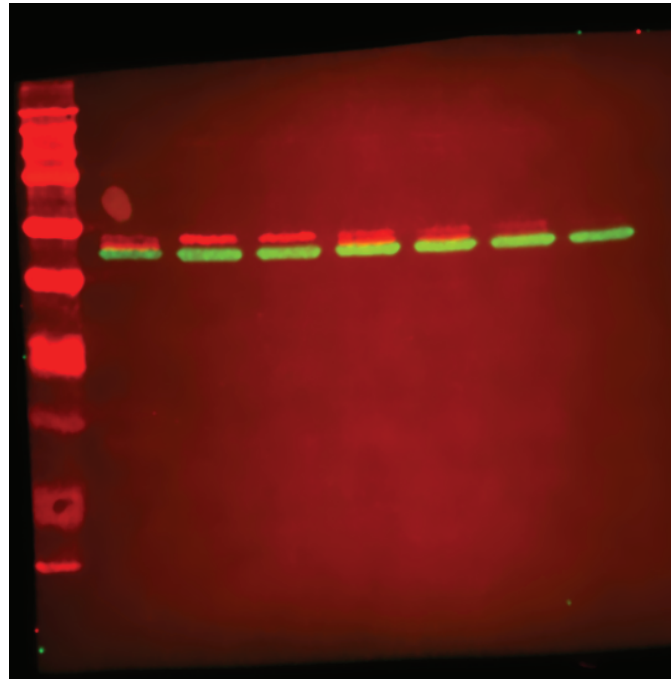

$\alpha$ SQR (red)  
 $\alpha$ actin (green)

Supplement: Extended Data Fig. 3 — Source data [file 41589_2022_1145_MOESM14_ESM.zip › Ex.Fig3/WB_7_Ex.Fig.3G.pdf]
